# Association of Common Variants of *TNFSF13* and *TNFRSF13B* Genes with CLL Risk and Clinical Picture, as Well as Expression of Their Products—APRIL and TACI Molecules

**DOI:** 10.3390/cancers12102873

**Published:** 2020-10-06

**Authors:** Monika Jasek, Agnieszka Bojarska-Junak, Maciej Sobczyński, Marta Wagner, Sylwia Chocholska, Jacek Roliński, Dariusz Wołowiec, Lidia Karabon

**Affiliations:** 1 Laboratory of Genetics and Epigenetics of Human Diseases, Hirszfeld Institute of Immunology and Experimental Therapy, Polish Academy of Sciences, 53-114 Wroclaw, Poland; marta.wagner@hirszfeld.pl (M.W.); lidia.karabon@hirszfeld.pl (L.K.); 2 Department of Clinical Immunology, Medical University of Lublin, 20-093 Lublin, Poland; agnieszkabojarskajunak@umlub.pl (A.B.-J.); jacek.rolinski@umlub.pl (J.R.); 3 Department of Bioinformatics and Genomics, Faculty of Biotechnology, University of Wrocław, 50-383 Wroclaw, Poland; macsebsob@poczta.onet.pl; 4 Department of Haematooncology and Bone Marrow Transplantation, Medical University of Lublin, 20-081 Lublin, Poland; sylwia.chocholska@umlub.pl; 5 Department and Clinic of Haematology, Blood Neoplasm, and Bone Marrow Transplantation, Wroclaw Medical University, 50-367 Wroclaw, Poland; wolowiec@post.pl

**Keywords:** chronic lymphocytic leukaemia (CLL), APRIL (*TNFSF13*), TACI (*TNFRSF13B*), SNPs, susceptibility, expression

## Abstract

**Simple Summary:**

A growing body of evidence reported in literature suggests an important role of APRIL in the development and pathogenesis of chronic lymphocytic leukaemia (CLL), in particular elevated levels of soluble APRIL (sAPRIL) and overexpression of its mRNA have been noticed in CLL cells. Moreover, TACI expression has been found to improve the survival ability of CLL cells protecting them from apoptosis in vitro. On the other hand, there are evidence supporting the existence of a genetic predisposition to develop CLL. These data prompted us to investigate the influence of genetic variants of *TNFSF13* and *TNFRSF13B* on the expression level of proteins encoded by these genes and to investigate the association between these variants an CLL risk.

**Abstract:**

Interactions between APRIL (TNFSF13) and its receptor TACI (TNFRSF13B) are implicated in providing survival benefits for chronic lymphocytic leukaemia (CLL) cells. Here we explored the relationship between *TNFSF13* and *TNFRSF13B* SNPs and expression of APRIL and TACI molecules and performed extended case-control study to evaluate earlier observations. Expression of APRIL and TACI was detected by FACS for 72 and 145 patients, respectively, and soluble APRIL was measured by ELISA in plasma of 122 patients. Genotypes were determined in 439 CLL patients and 477 control subjects with TaqMan Assays or restriction fragment length polymorphism (RFLP). The rs4968210GG genotype of *TNFSF13* was associated with a lower percentage of CD19^+^APRIL^+^ cells in CLL patients when compared to (AA + GA) genotypes (*p*-value = 0.027). Homozygosity at rs11078355 *TNFRSF13B* was associated with higher CD19^+^ TACI^+^ cell percentage in CLL patients (*p*-value = 0.036). The analysis of extended groups of patients and healthy controls confirmed the association of *TNFSF13* rs3803800AA genotype with a higher CLL risk (OR = 2.13; CI95% = 1.21; 3.75; *p*-value = 0.007), while the possession of *TNFRSF13B* rs4985726G allele (CG + GG) genotype was associated with lower risk of CLL (OR = 0.69; CI95% = 0.51; 0.95; *p*-value = 0.02). Genetic variants of *TNFSF13* and *TNFRSF13B* may have an impact on APRIL and TACI expression and may be considered as possible CLL risk factors.

## 1. Introduction

Chronic lymphocytic leukaemia (CLL) is the commonest leukaemia of adults in the Western World [1] with an average incidence around 0.06% among Europeans and citizens of the United States of America [2]. CLL is two-times more frequent among men and mainly occurs in elderly individuals with a median age of diagnosis ranging from 67 to 72 years [1,2,3].

The characteristic feature of CLL is clonal expansion of mature, usually CD5^+^ B lymphocytes that slowly accumulate in the peripheral blood, bone marrow, and lymphoid tissues mainly due to alternation in apoptosis mechanism [1,2,3,4].

CLL is a highly heterogeneous disease considering its biology and clinical course [1] which may partially explain the differences in the aggressiveness of CLL among patients as well as their response to therapy [1,3]. Apart from recurrent somatic mutations and recurrent somatic copy number variations (e.g. *NOTCH1*, *POT1*, *PTPN11*, *TP53*, *ATM*) [3], miRNA alternations (e.g. *mir-155*, *mir-15a*) and other epigenetic changes, the genomic landscape of CLL also includes chromosomal alternations such as del(13q), del(11q), trisomy 12 (+12), and del(17p13) [2]. The aberration of chromosome 17, especially in the locus of tumour suppressor *TP53*, is identified in 5% to 10% of newly diagnosed CLL patients, but can be detected even in 30% of relapsed/refractory cases and its prevalence can increase throughout the duration of the disease course [5].

Several lines of evidence also support the existence of a genetic predisposition responsible for CLL development [5]. The risk of developing CLL for the relatives of CLL patients is much higher (2- to 8-fold increase) than in the general population [5], and about 10% of patients with diagnosed CLL have the relatives with this disease [6]. The contribution of genetic factors to CLL risk has been also confirmed by the results of linkage and genetic association studies [7]. Recently, it has been reported that CLL risk loci occur frequently in CLL-specific regulatory elements such as enhancers as well as regulatory elements differently regulated during CLL and B cell development [8].

The tumour microenvironment plays an important role in CLL, since neoplastic cells rely on survival factors such as B-cell activating factor (BAFF; TNFSF13B) and proliferation-inducing ligand (APRIL; TNFSF13), provided, inter alia, by non-neoplastic nurse like cells (NLCs) [2,9]. The gene signature analysis of CLL cells derived from a distinct CLL microenvironment i.e. peripheral blood, bone marrow, and lymph nodes has shown that BAFF/APRIL related genes were enriched in LN-CLL [10]. BAFF and APRIL provide survival signals to CLL cells through interaction with their receptors. B-cell maturation antigen (BCMA, TNFRSF17) as well as transmembrane activator and calcium-modulator and cyclophilin ligand (CALM) interactor (TACI, TNFRSF13B) are common receptors for both BAFF and APRIL. BAFF also interacts exclusively with the BAFF receptor (BAFF-R, TNFRSF13C), and APRIL binds additionally to heparan sulphate proteoglycans (HSPGs) [11,12].

In our preliminary study we investigated the impact of genetic variants of *TNFSF13* (*APRIL*), *TNFSF13B* (*BAFF*), *TNFRSF17* (*BCMA*), *TNFRSF13C* (*BAFF-R*) and *TNFRSF13B* (*TACI*) genes on CLL risk [13] as well as *TNFSF13B* variants on expression levels of intracellular and soluble forms of BAFF [14], here we focused on investigating the relationship between *TNFSF13* (*APRIL*) and *TNFRSF13B* (*TACI*) genetic variants and CLL risk and expression of APRIL and TACI molecules.

APRIL is a member of the tumour necrosis factor superfamily (TNFSF) that is expressed as a type II transmembrane protein and encoded by *TNFSF13* gene (17p13.1) [15]. APRIL normally occurs in a soluble form as it is cleaved intracellularly in the Golgi apparatus by a furin convertase before it is released from the cell [12]. As was mentioned above, APRIL binds with high affinity to two receptors belonging to the TNF family receptors (TNF-R) BCMA and TACI [12]. APRIL may also bind HSPGs [12]. This interaction is important as it may be necessary for soluble APRIL (sAPRIL) to present itself to its receptors [16] and to pass productive signals through the TACI and the BCMA receptors [15]. APRIL has been shown to be a critical factor influencing the survival of activated B cells and plasmablasts in bone marrow [16]. With regards to that, elevated levels of sAPRIL and overexpression of its mRNA have been noticed in CLL cells [16]. In the study by Bojarska-Junak and colleagues [17], a higher median percentage of peripheral blood CD19^+^ cells with an intracellular APRIL expression and higher level of sAPRIL in plasma of CLL patients has been demonstrated [17].

TACI receptor, encoded by the *TNFRSF13B* (17p11.2) gene, plays an important role in controlling such processes as: an isotype switch, a B cells homeostasis, and a T-cell independent B cell antibody response [11]. Specifically, the APRIL-TACI interaction has been shown to be pivotal to a class switch recombination (CSR) to IgA [11]. Additionally, some studies indicated that a high TACI expression improves the survival ability of CLL cells protecting them from apoptosis in vitro [18,19].

As mentioned above, a considerable body of evidence indicates that APRIL-TACI interactions play an important role in CLL. The results of our earlier observations suggest that the genetic polymorphisms of *TNFSF13* and *TNFRSF13B* genes may constitute the CLL susceptibility variants [13]. Taking the above into consideration, in our present study we evaluated the impact of *TNFSF13* and *TNFRSF13B* SNPs on APRIL and TACI molecules expression in CLL cells. Additionally, we carried out an extended case-control study for *TNFSF13* and *TNFRSF13B* SNPs in CLL patients and control subjects to evaluate previously observed associations [13].

## 2. Results

### 2.1. Copy Number Variations

The *TNFSF13* and *TNFRSF13B* genes are located on chromosome 17 (17p13.1 and 17p11.2, respectively), which is frequently deleted in CLL. We determined a single copy of *TNFSF13* gene in 4.67% and a single copy of *TNFRSF13B* gene in 2.97% of CLL patients. All samples for which a single copy of *TNFSF13* and/or *TNFRSF13B* gene were detected, were excluded from all analyses conducted in this study.

### 2.2. Genotyping Results

As previously described in detail in our earlier publication [14], we investigated nine SNPs of *TNFSF13* and *TNFRSF13B* genes [13], on enlarged (relative to our previous study [13]) groups of CLL patients (N = 439) and control subjects (N = 477). Deviation from Hardy Weinberg Equilibrium (HWE) was noticed only for one out of nine examined SNPs, namely for the rs3803800G>A in the CLL group (*p*-value = 0.013; *f* = 0.12; CI95% = 0.02; 0.22) (Table 1; Supplementary Tables S1 and S2).

#### 2.2.1. TNFSF13 Genetic Variants and Susceptibility to CLL

The *TNFSF13* genotype distribution for CLL patients and controls is presented in Table 1 and Supplementary Table S1. Similarly to our prior study [13], we observed a significant difference in genotype distribution among CLL and healthy subjects for rs3803800G>A (a missense variant of *TNFSF13* Ser96Asn, exon 2, and *TNFSF12-TNFSF13 (TWE-PRIL)* Ser176Asn, exon 7) (χ^2^_d*f* = 2_ = 8.56; *p*-value = 0.0139). We noticed here a higher frequency of homozygotes AA among CLL patients than in controls (previously: 8.0% vs. 3.4%; here 8.2% vs. 4.0%). This two times higher frequency of homozygotes AA in patients correlated with the lower frequency of heterozygotes GA among patients (32.3%) when compared to healthy control (HC) (37.5 %). The OR value for homozygotes AA (OR = 2.00; CI95% = 1.13; 3.56), was the same as that observed in our earlier work [13]. Next, we compared the AA homozygotes to the G allele carriers (GG and GA) and confirmed that the AA genotype was associated with two times higher risk of CLL (OR = 2.13; CI95% = 1.21;3.75; *p*-value = 0.0073). We did not find association between CLL risk and other genetic variants of *TNFSF13* investigated here.

#### 2.2.2. TNFRSF13B Genetic Variants and Susceptibility to CLL

As for the *TNFRSF13B* gene, the genotype distribution of five examined SNPs for CLL patients and controls are presented in Table 1 and Supplementary Table S2. In our earlier study [13] we pointed to rs4985726C>G (intron 1 variant) as a potential CLL risk variant. Here, we confirmed our previous observation that CG heterozygotes were more frequent in HC than in the patient group (24.5% vs. 17.8%, respectively) and this increase resulted from the decrease in the frequency of CC homozygotes in the control group (74.0% vs. 80.4%). Additionally, we observed that subjects with CG genotypes had about a 1.5 (OR = 0.67) lower risk of CLL than subjects with CC genotypes. This observation may suggest a protective role of allele G, since carriers of allele G (CG+GG) showed a lower risk of CLL (OR = 0.69; CI95% = 0.51; 0.95; *p*-value = 0.0213). We did not find an association between CLL risk and other genetic variants of *TNFRSF13B* investigated here.

### 2.3. Relationship between TNFSF13 Genetic Variants and APRIL Intracellular Expression in CLL Cells

Next we analysed the relationship between the investigated *TNFSF13* SNPs and an intracellular expression of APRIL determined by a mean fluorescence intensity (MFI) and a percentage (%) of CD19^+^APRIL^+^ cells derived from CLL patients (Supplementary Tables S3, S4, respectively). We observed an association between rs4968210G>A SNP (rs4968210 variant constitutes G to A substitution in intron 5 of TNF-like weak inducer of apoptosis (*TNFSF12, TWEAK*) transcript as well as of *TNFSF12-TNFSF13 (TWE-PRIL*) transcripts and the percentage of CD19^+^APRIL^+^ CLL cells. Patients with the rs4968210GG genotype had a lower average percentage of CD19^+^APRIL^+^ cells in comparison to patients with the rs4968210GA or rs4968210AA genotypes (6.17 vs. 12.29 vs. 12.07, respectively, *p*-value = 0.0271) (Supplementary Table S4). Based on this, it can be assumed that A allele is associated with a higher average percentage of CD19^+^APRIL^+^ CLL cells.

### 2.4. Relationship between TNFSF13 Genetic Variants, Soluble APRIL, Rai Stage, and IgA Levels

In the next step of our study, we examined the association between sAPRIL plasma levels and genotypes of investigated *TNFSF13* SNPs. We did not observe associations between any of the SNPs examined here and plasma levels of sAPRIL (Supplementary Table S5). Additionally, in our group of CLL patients there was no association between sAPRIL plasma levels and Rai stages. Average levels of sAPRIL in patients in Rai stage 0,1,2,3,4 were 31.33,25.84,28.3,29.06,27.02 ng/ml, respectively (Supplementary Figure S1).

**Figure 1:**
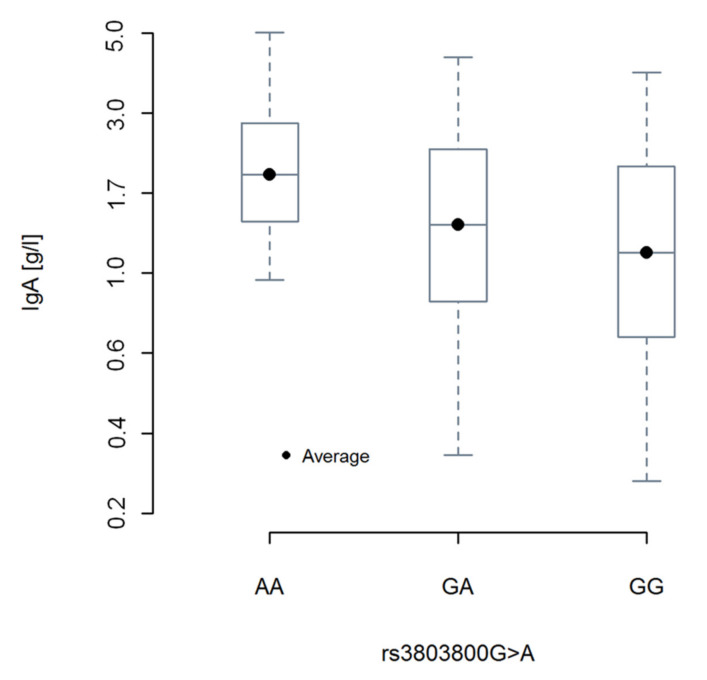
Levels of plasma IgA (g/L) in groups of patients according to the rs3803800G>A genotype. Box-and-whisker plots in standard manner with expected value as central points, 1st and 3rd quartiles and min-max values. The average levels of IgA (g/L) in groups of patients according to rs3803800G>A genotype AA, GA, and GG are 1.89, 1.36, and 1.14, respectively. Pearson’s correlation coefficient between these averages and genotypes is r*_altering_* = –0.987. It means that there is a strong linear relationship between rs3803800G>A genotype and expected value of IgA, but this coefficient ignores variability of IgA (g/L) within groups with a particular genotype. When this additional within-group variation is incorporated, the effect size correlation between genotype and IgA is r_effect size_ = –0.140. Observed differences between average levels of IgA in three groups are significant with *F* = 4.2475; *p* = 0.0178.

As association between the *TNFSF13* rs3803800AA genotype and serum IgA levels has been observed by other groups [20,21,22], we decided to investigate the relation between genotypes of rs3803800G>A and IgA levels in CLL patients (Supplementary Table S6). As presented in Figure 1, we noticed the highest plasma levels of IgA for CLL patients being homozygotes AA at rs3803800 and we observed a linear trend of IgA average levels declining as number of A alleles decreases.

### 2.5. Relationship between TNFRSF13B Genetic Variants and TACI Expression and Rai Stage

Analysis of the impact of *TNFRSF13B* variants on TACI expression revealed association between genotypes of rs11078355A>G (a synonymous variant, Ser277Ser; exon 5) and expression of TACI receptor (MFI and % of CD19^+^TACI^+^ leukemic cells). The genotype rs11078355AA was associated with lower average values of TACI (MFI) (34.72) as compared to AG (43.40) and GG (41.90) (AA vs AG + GG *p*-value = 0.0185) (Table 2). Moreover, we observed higher average percentage of leukemic cells with TACI membrane expression in both types of homozygotes (AA 12.13%; GG 15.35%), and a considerably lower expression in the case of heterozygotes (7.12%) (AG vs. AA+GG *p*-value = 0.036) (Table 2).

**Figure 2:**
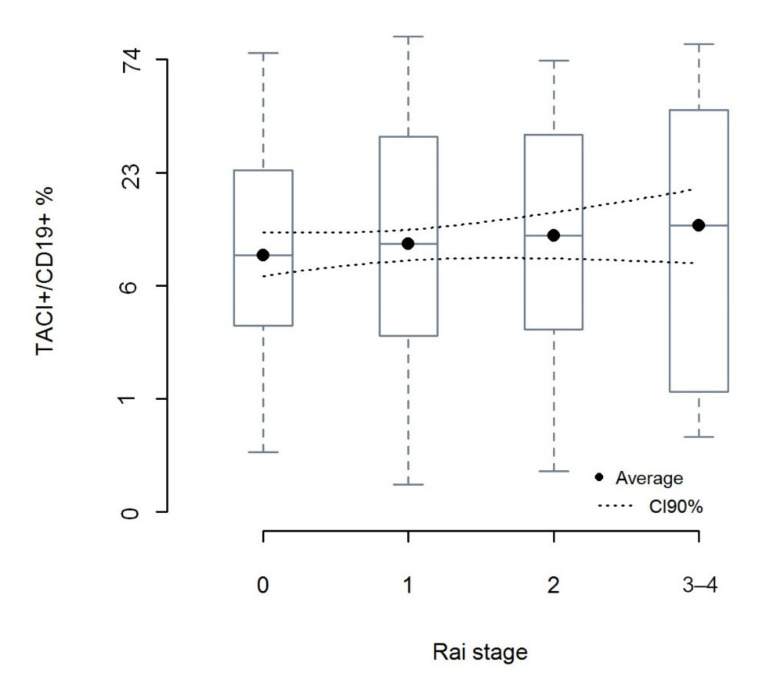
The percentage of CD19^+^TACI^+^ leukemic cells in groups of CLL patients according to the Rai stage. Box-and-whisker plots in standard manner with expected value as central points, 1st and 3rd quartiles and min-max values. Difference between groups tested with ANOVA with linear contrasts. Although in case of a patient there can be 0,1,2,3 or 4 Rai stage score, but in case of any group of patients there is always a mean of such stages, which can take any value between 0 and 4. Dot lines bounds 90% confidence interval for the mean of CD19^+^ TACI^+^% in a group of patients with particular mean of Rai stage. For ANOVA table and additional information please see Supplementary Table S7.

We observed a linear relationship between the averages of CD19^+^TACI^+^ percentage of leukemic cells and an increase of Rai stage (r_alerting_ = 0.998), however due to a strong variability of CD19^+^TACI^+^ within groups, the effect size of association between Rai stage and percentage of CD19^+^TACI^+^ cells was low r_effect size_ = 0.084 (Figure 2; Supplementary Table S7).

### 2.6. Prediction of Functional Effects for: TNFSF13 rs3803800G>A, rs4968210G>A and TNFRSF13B rs4985726C>G, and rs11078355A>G

Since elucidating the function of risk variants and variants associated with phenotypic features is an important step towards a better understanding of the biological processes involved in disease development and outcomes, we used publicly available sources, described in the in silico analysis in the Material and Methods section, to examine a potential functional relevance of rs3803800G>A, rs4968210G>A, rs4985726C>G and rs11078355A>G genetic variants. The detailed results of this analysis can be found in Supplementary Data 8.

## 3. Discussion

The role of APRIL in malignancies has originally been shown in solid tumours and cancer cell lines and was related to the stimulation of neoplastic cell proliferation both in vitro and in vivo [15]. Further studies demonstrated an involvement of APRIL, inter alia, in lymphoid malignancies, including CLL, autoimmune diseases such as systematic lupus erythematosus [23], Sjörgen syndrome [15], and IgA nephropathy [20,21,24]. A growing body of evidence reported in literature as well as the results of our previous study [13] suggesting an important role of APRIL in the development and pathogenesis of CLL, prompted us to investigate the correlation between variants of *TNFSF13* and *TNFRSF13B* genes and expression level of APRIL and TACI molecules in CLL patients.

In the first stage of our research we analysed the relationship between *TNFSF13* and *TNFRSF13B* SNPs and expression of APRIL and TACI molecules. Next, we performed genotyping of selected *TNFSF13* and *TNFRSF13B* SNPs on additional CLL patients and control subjects to confirm our previous observations [13] and to look for new associations.

In the present study, we confirmed our earlier observation that the homozygotes rs3803800AA of *TNFSF13* had two times higher risk of development of CLL than the carriers of G allele (GG + GA).

We did not find association between rs3803800 and APRIL expression, however our in silico analysis with application of ENCODE [25,26] revealed that rs3803800 localized to a candidate Cis Regulatory Element (cCRE) accession number EH38E1844485 (hg38), which was shown to have a proximal enhancer like signature and was associated with *TNFSF13* expression (Supplementary Figure S2B,C, respectively). In order to evaluate if this variant may affect *TNFSF13* expression in other tissues we performed an additional examination with application of GTEx multi-tissue eQTLs analysis [27]. The rs3803800 SNP appeared to be significantly associated with APRIL expression in many tissues (Meta-Analysis RE2: *p*-value = 5.2 × 10^−55^). This analysis indicated that the minor allele A of rs3803800 (reference allele in GTEx) was associated with higher APRIL expression in the majority of tissues (Supplementary Figure S2D). This observation may partially explain the association demonstrated by us between the rs3803800AA genotype and higher susceptibility to CLL, since this genotype may be responsible for a predisposition to the higher production of APRIL. To the best of our knowledge, we are the first to report such an association, thus our results need to be confirmed by other groups.

Kawasaki et al. [23] described association between rs3803800A allele and a risk of systemic lupus erythematosus (SLE) [23]. It may suggest contribution of the *TNFSF13* gene and its product to the pathogenesis of this disease, particularly when taking into consideration studies reporting increasing risk of haematological malignancies, particularly non-Hodgkin lymphoma (NHL) in SLE [28]. The strongest association has been found between SLE and diffuse large B cell lymphoma (DLBCL) [28]. The exact mechanism responsible for the development of lymphomas in SLE patients has not been established yet, however involvement of some cytokines, including APRIL, has been proposed as one of the possible explanations. The data regarding contribution of APRIL to SLE development are limited, but increased levels of sAPRIL were found in subgroups of SLE patients. Moreover, in patients with SLE who developed DLBCLs, higher mean percentage of cell expressing APRIL in comparison to patients diagnosed with DLBCL without SLE was observed [28].

The rs3803800AA genotype of *TNFSF13* was also found to be associated with susceptibility to IgA nephropathy (IgAN) [20,24] and with higher serum IgA levels in comparison to GG and GA genotypes, but not with APRIL serum levels [20]. Similarly, we found the highest plasma levels of IgA for CLL patients being homozygotes AA at rs3803800. The fact that rs3803800AA genotype was associated with higher IgA levels in CLL and IgA nephropathy suggests that this genotype may be associated with higher IgA production in general, regardless of ethnicity or disease. It will be interesting to examine in the future, if CLL patients with the *TNFSF13* rs3803800AA genotype are more prone to develop IgA nephropathy.

Here, we also noted an association between rs4968210 allele A of *TNFSF13* and average percentage of CLL cells with intracellular expression of APRIL. Since rs4968210, according to ENCODE [25,26], is located not far from, cCRE EH38E1844474 (hg38) with a predicted distal enhancer like signature associated with B cell biology and *TNFSF12-TNFSF13* expression, different genotypes of rs4968210 or SNPs in Linkage disequilibrium (LD) [29,30] with this variant may potentially affect APRIL expression (Supplementary Figure S3A–C). The GTEx multi-tissue eQTLs analysis [27] in terms of impact of this variant on *TNFSF13* and *TNFSF12* expression seemed to confirm our observation (Supplementary Figure S3D,E). Rs4968210 turned out to be significantly associated with *TNFSF12* expression in many tissues (Meta-Analysis RE2: *p*-value = 1.2 × 10^−94^). This analysis indicated that the rs4968210G allele (reference allele in GTEx) was associated with higher *TNFSF12* expression in the majority of tissues for which the data were available. However, in EBV-transformed lymphocytes an opposite effect was observed, unfortunately this association was not significant. This is in line with our observation that patients with rs4968210GA and rs4968210AA genotypes had higher average percentage of CD19^+^APRIL^+^ CLL cells. Rs4968210 was also associated with *TNFSF13* eQTLs expression in multiple tissues (Meta-Analysis RE2: *p*-value = 1.9 × 10^−11^). The result of this analysis suggests that rs4968210 may be associated with allele specific expression which seems to be tissue specific (Supplementary Figure S3E). One potential explanation of the observed effect is that the examined SNPs are responsible for creation or disruption of TF binding sites. However, other mechanisms by which SNPs in regulatory regions may contribute to the disease, such as changes in chromatin accessibility or DNA-looping has to be also considered [31]. Further studies are needed to evaluate our observation and to establish the mechanism responsible for this phenotypic change.

The *TNFSF13* rs3803800G>A SNP and the *TNFRSF13B* rs11078355A>G investigated in our study, were previously described in a context of biological response to atacicept [32]. The atacicept is a human recombinant soluble fusion protein [33] consisting of the extracellular (ligand binding) domain of TACI receptor joined to Fc portion of IgG1 [12,33]. This compound plays the role of a “decoy receptor” for BAFF and APRIL [34] by binding soluble and membrane-bound forms of BAFF and APRIL [12,32,33] and it is being evaluated as a treatment option for SLE patients [33,35].

Kofler et al. investigated the relations between biological activity of atacicept and genetic variants of some of BAFF/APRIL axis genes and delivered preliminary data suggesting that *TNFSF13* rs3803800G>A and *TNFRSF13B* rs11078355A>G may be associated with response to atacicept treatment in CLL [32]. As for *TNFRSF13B* gene, it has been reported that homozygotes GG and AA at rs11078355A>G site more frequently presented stable disease or partial response to treatment whereas heterozygotes AG more frequently suffered from progressive disease [32]. We found that patients with rs11078355AA and rs11078355GG genotypes had higher average percentage of CD19^+^TACI^+^ cells in comparison to rs11078355AG heterozygotes. Additionally, we noticed that the rs11078355AA genotype was associated with slightly lower average values of TACI (MFI) in CLL cells (Supplementary Figures S4 and S5 present in silico predictions for rs11078355).

Given these data, we suppose that a better response of CLL patients being homozygotes at rs11078355A>G locus to atacicept [32] may arise out of higher frequency of cells expressing TACI receptor, since by blocking APRIL-TACI axis these cells may become more prone to apoptosis.

In another study, Guadagnoil and colleagues [15] developed APRIL antagonistic monoclonal antibodies with a view to be used for B-cell lymphomas treatment. They treated CLL cells with APRIL and observed that this stimulation induced survival advantage over time as a consequence of decreased apoptosis. The addition of APRIL antibodies completely reverted these effects. The authors concluded that their antibodies prevent APRIL-induced survival of neoplastic human B cells by interfering with TACI and BCMA APRIL receptors [15]. In a subsequent study, this group of researchers investigated the function of APRIL in the TCL1-Tg mouse model (transgenic model for CLL) and showed that “APRIL-mediated leukemic cell survival depended on TACI ligation” [36].

Above-mentioned studies indicated that the success of blocking of APRIL mediated survival signals depends inter alia on TACI expression. These observations pointed to the potential possibility of treatment with atacicept or APRIL antagonistic monoclonal antibodies (if considered to be evaluated in future clinical trials) in B-lymphoma and SLE patients with observed TACI receptor expression. In that context, our results suggest that in such a personalized approach the genotyping of *TNFRSF13B* rs11078355 may be considered as an important step for further investigation. As mentioned earlier, TACI expression on CLL cells is heterogeneous, and in the majority of cases the expression of this receptor is low or undetectable, which was observed in the current study and by other authors [15,17,19]. In line with our considerations, Mamara et al. [19] postulated that it will be preferable to test anti-APRIL treatment approaches only after the determination of TACI receptor expression on CLL cells [19].

Some studies indicated that high TACI expression improves the survival ability of CLL cells by protecting them from apoptosis in vitro [18,19], which in consequence may worsen the clinical course of the disease. Therefore, we decided to make additional analysis, and check whether we will be able to notice relationship between high TACI expression and Rai stage (Rai Staging System). We analysed the relationship between Rai stage and the average percentage of CD19^+^TACI^+^ and noted an increasing linear trend for the percentage of CD19^+^ TACI^+^ as Rai stage increases. This observation is in line with data showing that cells with membranous expression of TACI after binding of APRIL are protected from apoptosis.

As in our earlier study [13] we noticed that rs4985726C>G (Supplementary Figure S6) in *TNFRSF13B* gene may be considered as the risk factor of the development of CLL, with CC genotype being associated with a higher risk of disease development. Interestingly, in a study carried out by Enjuanes et al. [37], rs4985726C allele was part of a haplotype associated with a higher risk of CLL (rs4985726C; rs12051889C; rs4985694G; rs3818716A; OR = 1.29; CI95% = 1.07; 1.55; *p* = 0.006) [37].

It has been mentioned earlier that CLL cells largely depend upon the microenvironment which provide CLL cells with survival factors such as APRIL that provide survival signals through its receptors. Recently, the TACI-APRIL interaction has been studied in multiple myeloma (MM) cells and it has been demonstrated for regulatory T cells (Tregs) that this interaction may contribute to the immunosuppressive microenvironment of MM. Tregs expressed significantly higher levels of TACI receptors [38]. Hence, it has been postulated that APRIL may promote survival and proliferation of Tregs [38]. Furthermore, it has been also shown that TACI-mediated APRIL signalling had impact on an increased number of regulatory B cells (Bregs) from the bone marrow of MM patients as well as on higher production of IL-10 by Bregs [38]. Collectively, these results imply that APRIL may play a role in establishing an immunosuppressive tumour microenvironment in bone marrow of MM patients [38]. Taking the above into consideration, it can be anticipated that TACI-APRIL interaction may also contribute to the immunosuppressive tumour microenvironment in CLL, and if this happens, CLL patients with genotypes associated with higher expression of APRIL and/or TACI may progress to more aggressive forms of CLL. This issue seems to be worth further investigation.

In our earlier studies we also investigated associations between genetic variants of other members of BAFF/APRIL system, BAFF, BAFF-R, and BCMA. We did not find association between investigated SNPs of *BCMA* and risk of CLL, which was described in our previous publication [13] and confirmed on additional CLL patients (N = 439) and control subjects (N = 477) (Supplementary Table S8). We also did not confirm the previously observed interaction between rs951428 of *BAFF* and rs11570136 of *BCMA* and the age of CLL diagnosis on enlarged groups [13]. As far as BAFF and BAFF-R are considered, we described a possible association between rs9514828 and rs1041569 of *BAFF* as well as rs61756766 of *BAFF-R* and CLL risk. The genetic variants of *BAFF* investigated by us had no impact neither on plasma BAFF level nor BAFF intracellular expression by PB CD19^+^ CLL cells [14].

The results of our present study indicate that genetic variants of *TNFSF13* and *TNFRSF13B* genes may be considered as potential risk factors of CLL and that these variants may have an impact on APRIL and TACI molecules expression. We believe that our results expand current knowledge about the genetic background of CLL. Despite this fact, some limitations of our study ought to be discussed. Firstly, our case-control study was performed on a limited number of subjects taking into consideration the known low frequency of some genotypes. Secondly, some of the data analyzed by us, like APRIL and TACI molecule expression, plasma sAPRIL, or IgA levels, were not available for all samples. Thirdly, we cannot exclude the possibility that associations observed here for some of investigated SNPs may be related to other SNPs being in LD with genetic variants examined here. Due to the presented limitations our results have to be confirmed in future studies carried out on larger groups of patients and controls enrolled from populations of different ethnicities.

Collectively, the findings of our previous studies and the results presented here suggest that genetic variants of BAFF/APRIL system genes may constitute CLL risk factors. More research is needed in this field, particularly to assess association between additional SNPs and CLL risk as well as to elucidate the functional role of these variants in the context of B cell biology and CLL pathogenesis.

## 4. Materials and Methods

### 4.1. Study Groups

The present case-control study was carried out on a CLL patient group (N = 439) and a control group (N = 477). Detailed characteristic of these groups was presented in our previous publication [14]. Basic statistics of the main variables considered in the present study are available in Supplementary Table S9.

The study was approved by the Ethics Committee of the Medical University of Wroclaw (KB-312/2010 16 September 2010) and the Ethics Committee of the Medical University of Lublin (KE-0254/388/2003 18 December 2003). Written informed consent was obtained from all participants.

### 4.2. Selection of Single Nucleotide Polymorphisms and Genotyping

The criteria for SNP selection and genotyping were described earlier [13,14]. Briefly, the restriction fragment length polymorphism (RFLP) method was used for genotyping of the following SNPs of *TNFRSF13B* (rs4985726; rs8072293; rs11078355) and the allelic discrimination method with application of TaqMan SNP Genotyping Assays (Life Technologies, Carlsba, CA, USA) were used to determine the following SNPs: *TNFSF13* (rs3803800; rs4968210; rs11552708; rs6608); *TNFRSF13B* (rs11656106; rs12051889) [13]. Primer sequences, annealing temperatures and restriction enzymes are listed in Supplementary Table S10. Supplementary Table S11 contains a detailed list of assays used in this study. The Polymerase Chain Reactions (PCRs) were performed on T100™ Thermal Cycler (Bio-Rad, Hercules, CA, USA). TaqMan SNP Genotyping Assays were run on Applied Biosystems 7300 Real-Time PCR System (Thermo Fisher Scientific, Waltham, MA, USA).

### 4.3. In Silico Analysis

Several in silico tools were applied to predict possible functional implication for SNPs showing significant associations with CLL. The SNPinfo Web server (https://snpinfo.niehs.nih.gov/) was initially used to select SNPs with potential functional effect [39]. Additionally, the following tools were used to predict the potential functional impact of examined variants:

Exome Variant Server (https://evs.gs.washington.edu/EVS/) (Exome Variant Server, NHLBI GO Exome Sequencing Project (ESP), Seattle, WA (URL: http://evs.gs.washington.edu/EVS/) (2019/2020 accessed).

Ensembl Genome Browser (https://www.ensembl.org/Homo_sapiens/Info/Index);
dbSNP (https://www.ncbi.nlm.nih.gov/snp/);
Human Splicing Finder (http://www.umd.be/HSF/ [40].
HaploReg v4.1 (https://pubs.broadinstitute.org/mammals/haploreg/haploreg.php) [29,30].

HaploReg was additionally applied to assign potential functional SNPs in Linkage disequilibrium (LD) with variants examined here. ENCODE: Encyclopedia of DNA Elements (https://www.encodeproject.org/) [25,26] and Genome Browser (https://genome.ucsc.edu/) [41,42,43] were used to predict the association between SNPs investigated here and candidate Cis Regulatory Elements (cCREs). The Genotype-Tissue Expression Portal (GTEx) (V8) (https://gtexportal.org/home/) [27] was used for identification of potential associations between SNPs and gene expression levels (eQTLs; sQTLs), and to find out if any of the investigated SNPs affect APRIL or TACI expression in available tissues.

### 4.4. Copy Number Variations Determination

We examined Copy Number Variations (CNVs) of *TNFSF13* and *TNFRSF13B* genes as described in our preliminary study [13]. Copy Numbers were determined by qPCR with application of TaqMan Copy Number Assays (Thermo Fisher Scientific, Waltham, MA, USA) Hs02299109_cn for *TNFSF13* and Hs05487075_cn *TNFRSF13B* for TaqMan Human RNase P Assay was used as a copy number reference (Thermo Fisher Scientific, Waltham, MA, USA). All samples were run in triplicates. Reactions were performed according to the manufacturer’s protocol and the results were analysed with application of Applied Biosystems CopyCaller v2.0 Software (Thermo Fisher Scientific, Waltham, MA, USA).

### 4.5. Detection of sAPRIL Plasma Level and Intracellular Expression of APRIL in CLL Patients

Soluble APRIL plasma levels were measured by a commercial Human APRIL ELISA kit (Invitrogen, Carlsba, CA, USA) We followed the protocols recommended by the manufacturer. The ELISA Reader Victor 3 (PerkinElmer, USA) was used.

Mononuclear cells were separated by density gradient centrifugation with Biocoll Separating Solution (Biochrom, Harvard Bioscience, Holliston, MA 01746, US) for 25 minutes at 400 × *g* at room temperature. Interphase cells were removed, washed twice, and resuspended in phosphate-buffered saline (PBS).

For intracellular detection of APRIL, PBMC were stained with MAb against cell surface markers, i.e. CD19 FITC (Clone HIB19, BD Pharmingen, San Diego, CA, USA) (20 minutes at room temperature). Following membrane staining, cells were fixed and permeabilized with Cytofix/Cytoperm solution and Perm/Wash buffer (BD Pharmingen, San Diego, CA, USA ) according to the manufacturer’s instructions. Cells were then incubated for 30 mins at RT with PE-conjugated anti-APRIL MAb (Clone: A3D8, BioLegend, San Diego, CA, USA) or with an isotype control antibody. Finally, cells were washed and analyzed by flow cytometry directly after preparation. A FACSCalibur instrument (Becton Dickinson Franklin Lakes, NJ, USA) and CellQuest software (Becton Dickinson Franklin Lakes, NJ, USA) were used.

### 4.6. Detection of TACI Receptor Expression

TACI expression was determined by flow cytometry analysis with anti-TACI PE (Clone 165604, IgG_1_, R&D Systems, Inc. Minneapolis, MN, USA) as described previously [17].

### 4.7. Statistical Analysis

Departure from Hardy-Weinberg equilibrium was measured as

(1) $$f=\frac{p_{cc}-p^{2}{}_{c}}{p_{c}(1-p_{c})}$$

where $p_{c}$ and $p_{cc}$ are allele c and genotype cc frequencies. f<0 in case of deficiency of homozygotes, f>0 corresponds to deficiency of heterozygotes and f=0 when locus is in HWE. Difference between genotype distributions between patients and controls was tested with chi-squared test. Odds ratio and confidence interval were at 0.95 level for it was used as a measure of effect size. Basic statistics used to describe the main variables considered in the paper were median, first and third quartile, minimal and maximal observation. Median was used as a location parameter and S_n_ statistic was computing as a measure of variability [44].

(2) $$S_{n}=med\left\{med\left|x_{i}\right.\right.-\left.x_{j}\right|;j=1\ldots \left.n\right\}$$

This is the average difference between two randomly sampled observations. Analysis of Variance (ANOVA) and post hoc Fisher’s LSD test were used to test (a) differences between average levels of TACI MFI and % of TACI^+^ leukemic cells according to *TNFRSF13B* rs11078355A>G genotypes, (b) average levels of APRIL MFI in CD19^+^ CLL cells according to *TNFSF13* SNP genotypes, (c) average percentage of CD19^+^ APRIL^+^ CLL cells according to *TNFSF13* SNP genotypes and (d) average levels of plasma sAPRIL according to *TNFSF13* SNP genotypes. ANOVA with linear contrast was used to test differences in (a) IgA levels (g/l) in CLL patients according to rs3803800G>A genotype and (b) CD19^+^TACI^+^ percentage according to Rai stage. If necessary, data was transformed with the Box-Cox method. In case of ANOVA, F-distribution and *p*-value were estimated numerically based on 10000 bootstrap samples, if needed. Fligner-Killeen test was used to test hypothesis of variance homogeneity between compared groups. In case of ANOVA linear contrasts, a measure of effect size *r_effect size_* correlation statistic was used, where $r_{effect}{\;}_{size}=\sqrt{\frac{F_{contrast}}{F_{contrast}+df_{within}}}$ and *df_within_* are degrees of freedom within groups; *F_contrast_* has one degree of freedom in nominator. Statistic *r_effect size_* is interpreted as adjusted correlation between two variables (dependent and independent) after one adjusted for both any noncontrast between-group variation and within-group variation and *r_effect size_* ϵ $\left[0,1\right]$. All tested hypotheses were formulated a priori, during the study design [45]. For graphical presentation box-and-whiskers plots were used in standard manner with expected value as central point, 1st and 3rd quartiles, and min-max values.

## 5. Conclusions

In summary, the results obtained from this study provide considerable evidence that genetic variants of *TNFSF13* and *TNFRSF13B* genes may have an impact on CLL development. Additionally, our data on the association between *TNFRSF13B* rs11078355A>G genotypes and TACI membranous expression on CLL cells seems to be worthy of further investigation in diseases where blocking of APRIL-TACI interactions is considered as a treatment option. If such correlation will be confirmed, the genotyping of *TNFRSF13B* rs11078355A>G genetic variant may be used for selection of patients who will respond positively to such treatment.

## Figures and Tables

**Table 1:** Genotype distribution of *APRIL* (*TNFSF13)* rs3803800 polymorphism and *TACI* (*TNFRSF13B)* rs4985726 polymorphism in chronic lymphocytic leukaemia (CLL) patients and controls.

*APRIL* *TNFSF13* polymorphisms		Patients (N = 439)		Controls (N = 477)		OR	CI95%	Patients vs. Controls
		N	%	N	%			
**rs3803800 ^a^** **Asn96Ser**	GG	261	59.50	279	58.50	1 *		χ^2^_df = 2_ = 8.56 *p* = 0.014
	GA	142	32.30	179	37.50	0.85	0.64; 1.12	
	AA	36	8.20	19	4.00	2.00	1.13; 3.56	
**HWE**		*p* = 0.013		*p* = 0.154				
		*f* = 0.12		*f* = −0.07				
		CI95% = 0.02;0.22		CI95% = −0.15; 0.02				
***TACI*** ***TNFRSF13B*** **polymorphisms**		**Patients (N = 439)**		**Controls (N = 477)**		**OR**	**CI95%**	**Patients vs. Controls**
		**N**	**%**	**N**	**%**			
**rs4985726** **intron 1**	CC	353	80.40	353	74.00	1 *		χ^2^_df = 2_ = 6.30 *p* = 0.042
	CG	78	17.80	117	24.50	0.67	0.48; 0.92	
	GG	8	1.80	7	1.50	1.13	0.42; 3.06	
**HWE**		*p* = 0.135		*p* = 0.56				
		*f* = 0.07		*f* = −0.03				
		CI95% = −0.04;0.18		CI95% = −0.11;0.048				

Abbreviations: OR, odds ratio; CI, confidence intervals; HWE, Test for Hardy-Weinberg Equilibrium; *f*, departure from HWE, ^a^ G>A according to the frequency of alleles; * the reference group.

**Table 2:** Average values of TACI mean fluorescence intensity (MFI) and % of TACI^+^ leukemic cells according to rs11078355 genotypes.

TACI MFI	Genotype			F-test	*p*-value	LSD
**rs11078355**	**AA**	**AG**	**GG**			
average	34.72	43.40	41.90	4.102	0.0185	AA (AG.GG)
n	58	65	22			
**CD19^+^TACI^+^ %**						
**rs11078355**	**AA**	**AG**	**GG**			
average	12.13	7.12	15.35	3.41	0.0357	AG (AA.GG)
n	59	65	22			

The ANOVA test with different (homogeneous) groups identified based on Fisher’s LSD *post hoc* test was applied.

## Supplementary Materials

The following are available online at https://www.mdpi.com/2072-6694/12/10/2873/s1, Figure S1: Levels of sAPRIL in groups of CLL patients according to the Rai stage, Figure S2: In silico analysis—rs3803800 of *TNFSF13*, Figure S3: In silico analysis—rs4968210 of *TNFSF13*, Figure S4: Analysis of potential effects of rs11078355 A>G on splicing of *TNFRSF13B* gene, Figure S5: In silico analysis—rs11078355 of *TNFRSF13B*, Figure S6: In silico analysis—rs4985726 of *TNFRSF13B*, Table S1: Genotype distribution of the *APRIL* (*TNFSF13*; 17p13.1) polymorphisms in patients and controls, Table S2: Genotype distribution of the *TACI* (*TNFRSF13B*; 17p11.2) polymorphisms in patients and controls, Table S3: Average values of APRIL MFI in CD19^+^ CLL cells in relation to APRIL SNP genotypes, Table S4: Average percentage of CD19^+^ APRIL^+^ CLL cells according to APRIL SNP genotypes, Table S5: Average levels of plasma sAPRIL according to *APRIL* SNP genotypes, Table S6: ANOVA table with linear contrast for IgA levels [g/l] in CLL patients according to rs3803800G>A genotype, Table S7: ANOVA table with linear contrast for CD19^+^TACI^+^ percentage according to Rai stage. Table S8: Genotype distribution of the *BCMA* (*TNFRSF17*; 16p13.13) polymorphisms in patients and controls, Table S9: Basic statistics of the main variables considered in the paper, Table S10: Primer sequences, annealing temperatures and restriction enzymes used for PCR-RFLP genotyping, Table S11: TaqMan SNP Genotyping Assays used for genotyping with applying the allelic discrimination method, Supplementary Data 8: Prediction of functional effects for: *TNFSF13* rs3803800G>A, rs4968210G>A and *TNFRSF13B* rs4985726C>G and rs11078355A>G.
