# Supplementary material for: Association of Common Variants of TNFSF13 and TNFRSF13B Genes with CLL Risk and Clinical Picture, as Well as Expression of Their Products—APRIL and TACI Molecules
Source: Cancers (Basel). 2020 Oct 6;12(10):2873. doi: 10.3390/cancers12102873 (PMC7601931; doi:10.3390/cancers12102873)
Supplement: Supplementary file 1 [file cancers-12-02873-s001.pdf]

Supplementary Materials: Association of Common Variants of TNFSF13 and TNFRSF13B Genes with CLL Risk and Clinical Picture, as well as Expression of Their Products—APRIL and TACI Molecules

Monika Jasek, Agnieszka Bojarska-Junak, Maciej Sobczyński, Marta Wagner, Sylwia Chocholska, Jacek Roliński, Dariusz Wołowiec and Lidia Karabon

Supplementary Table S1. Genotype distribution of the *APRIL* (*TNFSF13;* 17p13.1) polymorphisms in patients and controls.

***APRIL TNFSF13* polymorphisms**		**Patients (N=439)**		**Controls (N=477)**		**OR**	**CI95%**	**Patients vs. Controls**
		**N**	**%**	**N**	**%**			
**rs11552708**	GG	385	87.70	417	87.42	1*		
	GA	53	12.10	57	11.95	1.01	0.68;1.50	$\chi^2_{df=2}$ =0.85
Gly67Arg	AA	1	0.20	3	0.63	0.46	0.07;3.16	p=0.782
**HWE**		p=0.833<br>*f*=-0.03<br>CI95%=-0.08;0.06		p=0.448<br>*f*=0.031<br>CI95%=-0.06;0.14				
**rs4968210**	GG	166	37.80	157	32.90	1*		$\chi^2_{df=2}$ =2.58
Intron 5	GA	198	45.10	237	49.70	0.79	0.59;1.05	p=0.275
	AA	75	17.10	83	17.40	0.86	0.58;1.25	
**HWE**		p=0.232<br>*f*=0.057<br>CI95%=-0.04;0.15		p=0.778<br>*f*=-0.020<br>CI95%=-0.11;0.07				
**rs6608**	CC	350	79.70	385	80.71	1*		
	CT	85	19.40	88	18.45	1.06	0.76;1.48	$\chi^2_{df=2}$ =0.14
3'UTR	TT	4	0.90	4	0.84	1.10	0.30;4.10	p=0.92
**HWE**		p=0.804<br>*f*=-0.02<br>CI95%=-0.10;0.07		p=0.947<br>*f*=-0.02<br>CI95%=-0.09;0.07				

Abbreviations : OR, odds ratio; CI, confidence intervals; HWE, Test for Hardy-Weinberg equilibrium; *f*, departure from HWE; * the reference group;

Supplementary Table S2. Genotype distribution of the *TACI* (*TNFRSF13B;* 17p11.2) polymorphisms in patients and controls.

TACI *TNFRSF13B* polymorphisms		Patients (N=439)		Controls (N=477)		OR	CI95%	Patients vs. Controls
		N	%	N	%			
**rs12051889** intron 1	CC	385	87.70	416	87.20	1*		$\chi^2_{df=2}$ =1.35 p=0.515
	CT	51	11.60	60	12.60	0.92	0.62;1.37	
	TT	3	0.70	1	0.20	2.52	0.37;17.16	
**HWE**		p=0.414<br>*f*=0.04<br>CI95%=-0.06;0.16		p=0.912<br>*f*=-0.03<br>CI95%=-0.08;0.045				
**rs8072293**[a] Thr27Thr	TT	211	48.10	236	49.48	1*		$\chi^2_{df=2}$ =0.25 p=0.881
	TC	195	44.40	204	42.77	1.07	0.82;1.40	
	CC	33	7.50	37	7.75	1.00	0.60;1.65	
**HWE**		p=0.209<br>*f*=-0.06<br>CI95%=-0.15;0.03		p=0.506<br>*f*=-0.036<br>CI95%=-0.12;0.053				
**rs11656106** intron 2	TT	206	46.90	238	49.90	1*		$\chi^2_{df=2}$ =0.87 p=0.649
	TC	194	44.20	197	41.30	1.14	0.87;1.49	
	CC	39	8.90	42	8.80	1.07	0.67;1.72	
**HWE**		p=0.576<br>*f*=-0.03<br>CI95%=-0.12;0.06		p=0.912<br>*f*=0.006<br>CI95%=-0.08;0.09				
**rs11078355** Ser277Ser	AA	157	35.80	158 33.10	158	1*		$\chi^2_{df=2}$ =2.03 p=0.362
	AG	206	46.90	246 51.60	246	0.84	0.63;1.12	
	GG	76	17.30	73 15.30	73	1.05	0.71;1.55	
**HWE**		p=0.554<br>*f*=0.028<br>CI95%=-0.07;0.12		p=0.185<br>*f*=-0.06<br>CI95%=-0.16;0.021				

Abbreviations : OR, odds ratio; CI, confidence intervals; HWE, Test for Hardy-Weinberg equilibrium; *f*, [a] T>C according to the frequency of alleles, * the reference group;

Supplementary Table S3. Average values of APRIL MFI in CD19+ CLL cells in relation to *APRIL* SNP genotypes.

APRIL SNP MFI APRIL+CD19+ CLL cells	Genotype			F-test	p-value	LSD*
**rs3803800**	**AA**	**GA**	**GG**			
average	81.49	86.76	80.80	0.124	0.883	(AA.GA.GG)
n	8	19	45			
**rs11552708**	**AA**	**GA**	**GG**			
average	59.63	91.63	81.71	0.36	0.702	(AA.GA.GG)
n	1	8	63			
**rs4968210**	**AA**	**GA**	**GG**			
average	79.6	75.49	92.32	1.159	0.32	(AA.GA.GG)
n	12	32	28			
**rs6608**	**CC**	**CT**	**TT**			
average	78.11	104.34	59.63	2.031	0.139	(CC.CT.TT)
n	57	14	1			

*The ANOVA test with different (homogeneous) groups identified based on Fisher's LSD *post hoc* test was applied.

Supplementary Table S4. Average percentage of APRIL$^+$ CD19$^+$ CLL cells according to *APRIL* SNP genotypes.

APRIL SNP % CD19$^+$ APRIL$^+$CLL cells	Genotype			F-test	p-value	LSD*
**rs3803800**	**AA**	**GA**	**GG**			
average	4.91	8.14	11.18	2.277	0.11	(AA.GA.GG)
n	8	19	45			
**rs11552708**	**AA**	**GA**	**GG**			
average	14.16	6.52	9.79	0.58	0.562	(AA.GA.GG)
n	1	8	63			
**rs4968210**	**AA**	**GA**	**GG**			
average	12.07	12.29	6.17	3.802	**0.0271**	**GG (AA.GA)**
n	12	32	28			
**rs6608**	**CC**	**CT**	**TT**			
average	10.08	6.9	14.16	0.774	0.465	(CC.CT.TT)
n	57	14	1			

*The ANOVA test with different (homogeneous) groups identified based on Fisher's LSD *post hoc* test was applied.

Supplementary Table S5. Average levels of plasma sAPRIL according to *APRIL* SNP genotypes.

APRIL SNP<br>sAPRIL level [ng/ml]	Genotype			F-test	p-value	LSD
**rs3803800**	**AA**	**GA**	**GG**			
average	31.80	25.69	29.84	0.699	0.499	(AA.GA.GG)
n	13	43	66			
**rs11552708**	**AA**	**GA**	**GG**			
average	-	23.28	29.09	0.86	0.354	(GA.GG)
n	0	11	111			
**rs4968210**	**AA**	**GA**	**GG**			
average	32.47	29.5	25.27	0.921	0.401	(AA.GA.GG)
n	20	62	40			
**rs6608**	**CC**	**CT**	**TT**			
average	29.56	25.5	14.1	1.056	0.351	(CC.CT.TT)
n	99	21	2			

*The ANOVA test with different (homogeneous) groups identified based on Fisher's LSD *post hoc* test was applied.

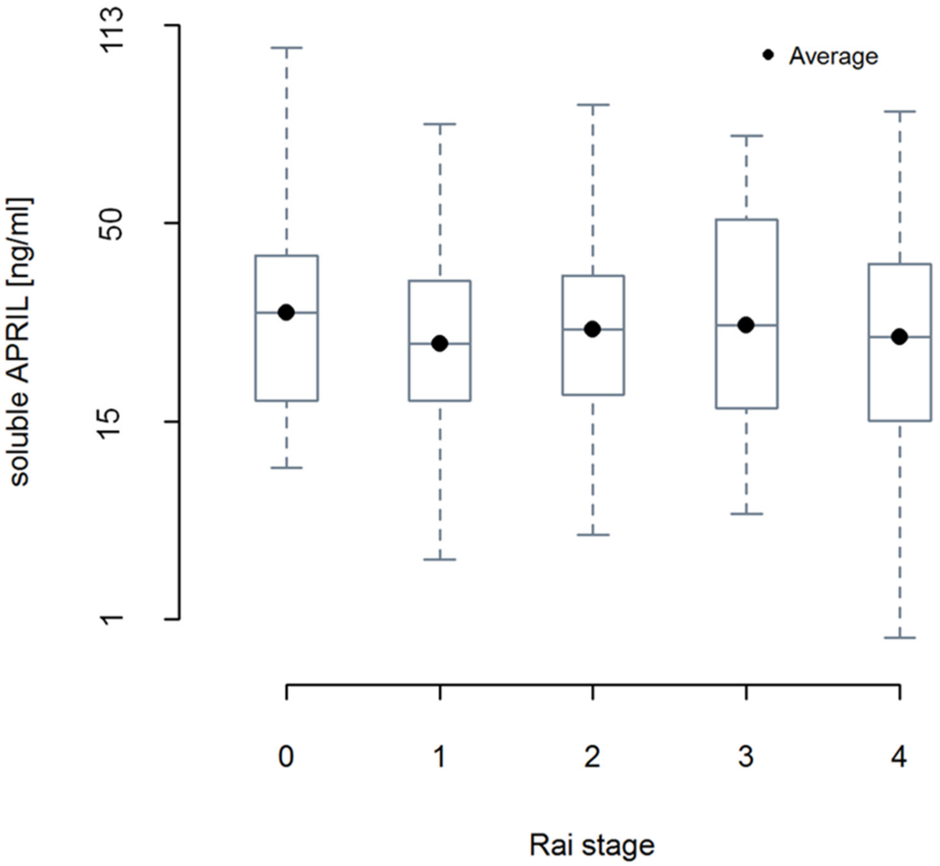

Supplementary Figure S1. Levels of sAPRIL in groups of CLL patients according to the Rai stage

Box-and-whiskers plots in standard manner with expected value as central points, 1st and 3rd quartiles and min-max values. Average levels of sAPRIL in groups of patients divided according to Rai stage (0, 1, 2, 3, 4) are 31.33, 25.84, 28.3, 29.06, 27.02 ng/ml, respectively. Figure shows average levels of sAPRIL as well as 1st and 3rd quartiles with min and max levels in each group of Rai stage. There is no relationship between Rai stage and sAPRIL level.

Supplementary Table S6. ANOVA table with linear contrast for IgA levels [g/l] in CLL patients according to rs3803800G>A genotype.

**Box-Cox transformation**	**rs3803800G>A**			
**λ = -0.096**	**AA**	**GA**	**GG**	
n	16	68	104	
**Mean**	0.616	0.304	0.130	
**Standard deviation**	0.417	0.72	0.803	
**Variance homogeneity**	$\chi^2_{df=3} = 6.087; p = 0.04767$ (Fligner-Killeen test)*			
**ANOVA table**				
**Source of variability**	**Degrees of freedom**	**Sum of squares**	**Mean of squares**	**F-test p-value**
Rai stage (linear contrast)	1	2.385	2.385	**F=4.247**
Error	186	104.45	0.562	**p=0.0178***

* F-distribution and p-value estimated numerically based on 10000 bootstrap samples.

Please note, that ANOVA was performed on data transformed with Box-Cox transformation (λ= -0.096). There is minor heterogeneity of variances between groups and correlation between means and SD, so the distribution of the F statistic and p-value were estimated with bootstrap method.

Supplementary Table S7. ANOVA table with linear contrast for $CD19^{+}TACI^{+}$ percentage according to Rai stage.

Box-Cox transformation $\lambda = 0.146$	Rai stage			
	**0**	**1**	**2**	**3-4**
n	50	43	40	13
Mean	2.54	2.73	2.88	3.06
Standard deviation	1.82	2.08	2.03	2.59
Variance homogeneity	$\chi^2_{df=3} = 4.384; p = 0.223$ (Fligner-Killeen test)			
**ANOVA table**				
**Source of variability**	**Degrees of freedom**	**Sum of squares**	**Mean of squares**	**F-test p-value**
Rai stage (linear contrast)	1	4.12	4.12	**F=1.0116**
Error	144	586.16	4.07	**p=0.3162**

Please note, that ANOVA was performed on data transformed with Box-Cox transformation ($\lambda = 0.146$). The average levels of $CD19^{+}TACI^{+}$ percentage of leukemic cells in groups of patients with Rai stage 0, 1, 2, and 3-4 are 8.7, 10.0, 11.1 and 12.5%, respectively. Pearson's correlation coefficient between these averages and Rai scores 0, 1, 2, 3 is $r_{altering} = 0.998$. It means that there is a strong linear relationship between Rai stage and expected percentage value of $CD19^{+}TACI^{+}$ cells, but this coefficient ignores a huge variability of (%) $CD19^{+}TACI^{+}$ cells within groups with particular Rai score. When this additional within-group variation is incorporated, the effect size correlation between Rai stage and (%) of $CD19^{+}TACI^{+}$ cells is $r_{effect\ size} = 0.084$. This within-group variability is a reason why mean of squares for the linear contrast in ANOVA is practically the same as mean of squares for error, thus $F_{1;144}=1.0116$; p=0.3162.

Supplementary Data 8. Prediction of functional effects for: TNFSF13 rs3803800G>A, rs4968210G>A and TNFRSF13B rs4985726C>G and rs11078355A>G.

Since elucidating the function of risk variants and variants associated with phenotypic features is an important step towards a better understanding of the biological processes involved in disease development and outcomes, we used publicly available sources, described below to examine a potential functional relevance of rs3803800G>A, rs4968210G>A, rs4985726C>G, and rs11078355A>G genetic variants.

rs3803800G>A, a missense variant of *TNFSF13* (Ser96Asn, exon 2) and *TNFSF12-TNFSF13* (Ser176Asn, exon 7) has been predicted to be a benign variant by PolyPhen2 (benign: 0.036) and as a tolerated substitution by SIFT (score:0.597, median: 2.93). According to HaploReg tool (v4.1) [1,2] this element is conserved and does not exist in LD with other SNPs (Supplementary Figure S2A).

Of note, *TNFSF13* is located in close proximity to the *TNFSF12* gene for another member of the *TNFSF*, namely the TNF-like weak inducer of apoptosis (TWEAK, TNFSF12). The adjacent localisation and the same transcriptional direction of these two genes enables the production of mRNA molecule (*TNFSF12-TNFSF13*) and protein (TWE-PRIL) consisted of the cytoplasmic/transmembrane and stalk domains of TWEAK and receptor binding domains of APRIL due to and intergenic splicing event.

Given the data provided by Bojarska-Junak et al. [3] showed a higher expression of APRIL mRNA and intracellular APRIL in peripheral blood (PB) $CD19^+$ leukemic cells than in PB $CD19^+$ cells isolated from the control group, we checked if rs3803800 localizes to any regulatory region by applying the ENCODE dataset [4-5]. One matching candidate is the Cis Regulatory Element (cCRE) accession number EH38E1844485 (hg38), which was shown for rs3803800G>A with a proximal enhancer like signature (Supplementary Figure S2B,C, respectively). This cCRE was associated with *TNFSF13* expression and according to ENCODE the IKZF1 transcription factor may bind within this cCRE, which was observed for GM12878 cell line. These data suggest that rs3803800 may potentially localize to regulatory region. The Supplementary Figure S2B,C presents rs3803800 surrounded by regulatory elements and TF binding sites (hg38). We have also performed an additional examination with application of GTEx [6] multi-tissue eQTLs analysis to see if this variant affect expression in other cells and tissues (Supplementary Figure S2D). The rs3803800 appeared to be significantly associated with *TNFSF13* expression in many tissues (Meta-Analysis RE2: p-value=$5.2x10^{-55}$). This analysis showed that the minor allele A of rs3803800 (reference allele in GTEx) is in the majority of tissues associated with higher *TNFSF13* expression.

rs4968210G>A variant constitutes G to A substitution in intron 5 of *TNFSF12-TNFSF13* transcript as well as of *TNFSF12* transcript. As described earlier, we observed that A allele is associated with a higher average percentage of $CD19^+APRIL^+$ CLL cells. Taking this into consideration we employed ENCODE [4-5] to check if this variant or any variant in LD with it (Supplementary Figure S3a) [1-2] localize to regulatory elements. According to ENCODE, rs4968210 variant does not overlap any cCRE, but is located 981 bp from cCRE EH38E1844474 (hg38) which is predicted to have a distal enhancer like signature inter alia in B cells and GM12878 (Supplementary Figure S3B and 3C) cell line and is associated with *TNFSF12-TNFSF13* mRNA expression. Additionally, another SNP in LD with rs4968210 (Supplementary Figure S3A) [1-2] namely rs12942590 is located within 221 bp distance of this cCRE (Supplementary Figure S3B and C). Accordingly, the transcription factors important for B cell biology such as EBF1, PAX5, SPI1, IKZF1, IKZF2, and BHLHE40 were shown to bind in this region (Supplementary Figure S3B and 3C).

The ENCODE data suggest that the impact of rs4968210 observed by us on phenotype of $CD19^+$ B cells in relation to APRIL may be associated with the fact that rs4968210 and rs12942590 are located near potential regulatory element associated with B cells biology and *TNFSF12-TNFSF13* expression. To further evaluate the association between rs4968210 and *TNFSF12-TNFSF13* expression we performed an additional analysis with application of GTEx multi-tissue eQTLs analysis to see if this variant affect expression in other cells and tissues (Supplementary Figure S3D). The rs4968210 turned out to be significantly associated with *TNFSF12* expression in many types of tissues (Meta -Analysis RE2: p-value=$1.2x10^{-94}$). This analysis indicates that the rs4968210G allele (reference allele in GTEx) is associated with higher *TNFSF12 (TWEAK)* expression. in the majority of tissues for that the data were available. However, in EBV-transformed lymphocytes an opposite effect can be observed, unfortunately this association was not significant. This is in line with our observation that patients with rs4968210GA and rs4968210AA genotypes had higher average percentage of $CD19^+APRIL^+$ CLL cells. The rs4968210 was also associated with *TNFSF13* eQTL expression in multiple tissues (Meta - Analysis RE2: p-value=$1.9x10^{-11}$). The result of this analysis suggests that rs4968210 may be associated with allele specific expression which seems to be tissue specific (Supplementary Figure S3E).

The Supplementary Figure S3A presents SNPs in LD with rs4968210 and Figure S3B,C present 4968210 and rs12942590 surrounded by regulatory elements and TF binding sites (hg38) in PB B cells as well as in GM12878 cell line, respectively.

rs4985726C>G variant is located in intron 1 of *TNFRSF13B* gene. According to HaploReg tool (v4.1) [1-2] this variant exists in LD with 10 intronic SNPs of *TNFRSF13B* and one missense rs34562254G>A (Pro251Leu) variant of *TNFRSF13B* (Supplementary Figure S4A). The rs34562254G>A (Pro251Leu) variant was predicted to be a possibly damaging variant by PolyPhen2 (Exome Variant server: 0.728; Ensembl: 0.476). Rs4985726C>G did not overlap with any cCRE both in PB B cells as well as in GM12878 cell line (Supplementary Figure S4B and S4C, respectively). Accordingly, we did not observe association between this variant and TACI expression in PB CLL cells. Similarly, analysis with GTEx portal [6] did not reveal significant eQTLs associated with rs4985726C>G. However, ENCODE [4-5] analysis for SNPs in LD with rs4985726 revealed data which may provide some potential explanation for the association of rs4985726 with the susceptibility to CLL risk observed by us. One of such SNPs, namely, rs57382045 did not overlap with any cCRE, however it is located 299 bp from EH38E1849614 cCRE (Supplementary Figure S4D,E). This cCRE was predicted to have distal enhancer-like signature in B cells with many TFs shown to bind in this region among others IKZF1 and IKZF2 (Supplementary Figure S4D). What is interesting, EH38E1849614 cCRE seems to be inactive (low DNase) in GM12878 cell line (Supplementary Figure S4E). The Supplementary Figure 4A presents SNPs in LD with rs4985726, Supplementary Figures S4B-E present rs4985726 and rs57382045, chromatin marks in B cells, and GM12878 cell line (hg38).

rs11078355A>G is a synonymous variant (exon 5, Ser277Ser) of the *TNFRSF13B* gene. According to HaploReg tool (v4.1) [1-2] this variant exists in LD with five SNPs (Supplementary Figure S6A). Analysis with application of Human Splicing Finder (HSF) [7] showed that this variant may potentially cause alternation of splicing, by affecting the binding site for 9G8 (also known as SRSF7) splicing factor (Supplementary Figure S5A). Additionally, our studies revealed an association between genotypes of rs11078355A>G and expression of TACI receptor on $CD19^+TACI^+$ leukemic cells. Given that, we checked if rs11078355 and/or any variant in LD with it localize to any cCREs with possible impact on B cell biology. We did not find such cCREs (Supplementary Figure S6B,C). Application of GTEx portal [6] did not reveal significant eQTLs and sQTLs for rs11078355A>G

associated with *TNFRSF13B* mRNA levels. The Supplementary Figure S6 displays rs11078355 and the surrounding *TNFRSF13B* gene region as well as SNPs in LD with rs11078355.

A

Query SNP: rs3803800 and variants with r^2 >= 0.8

chr	pos (hg38)	LD (r^2)	LD (D')	variant	Ref	Alt	AFR freq	AMR freq	ASN freq	EUR freq	SiPhy cons	Promoter histone marks	Enhancer histone marks	DNAse	Proteins bound	Motifs changed	NHGRI/EBI GWAS hits	GRASP QTL hits	Selected eQTL hits	GENCODE genes	dbSNP func annot
17	7559652	1	1	rs3803800	A	G	0.29	0.70	0.67	0.78		11 tissues	17 tissues	8 tissues		ERalpha-a,MZF1::1-4	4 hits	10 hits	4 hits	TNFSF13	missense

B

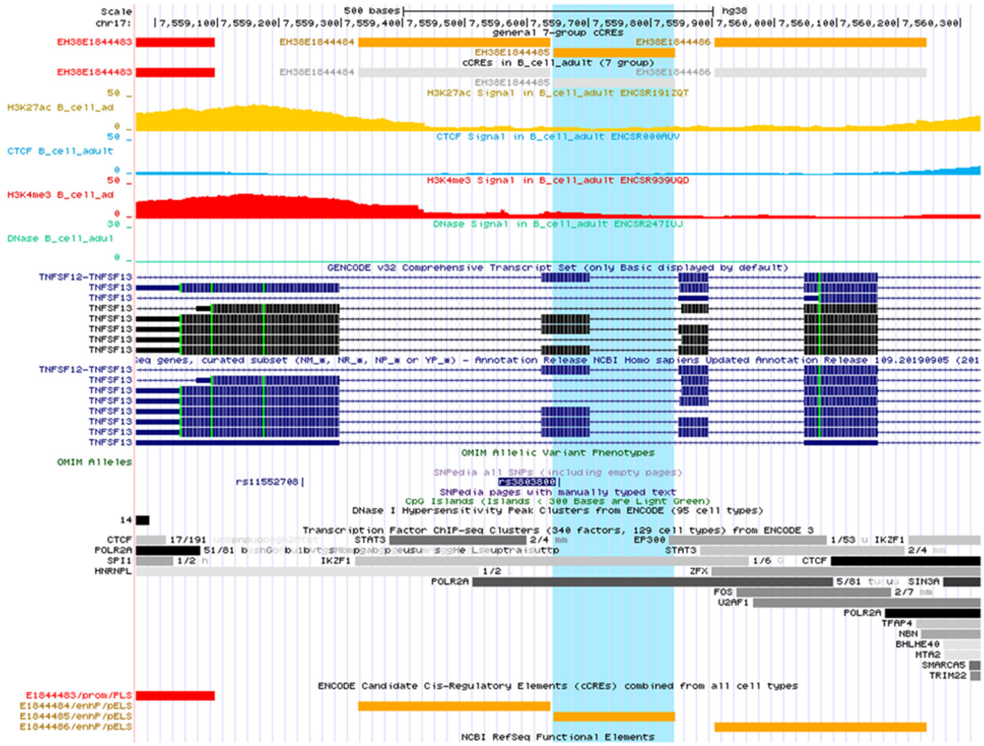

C

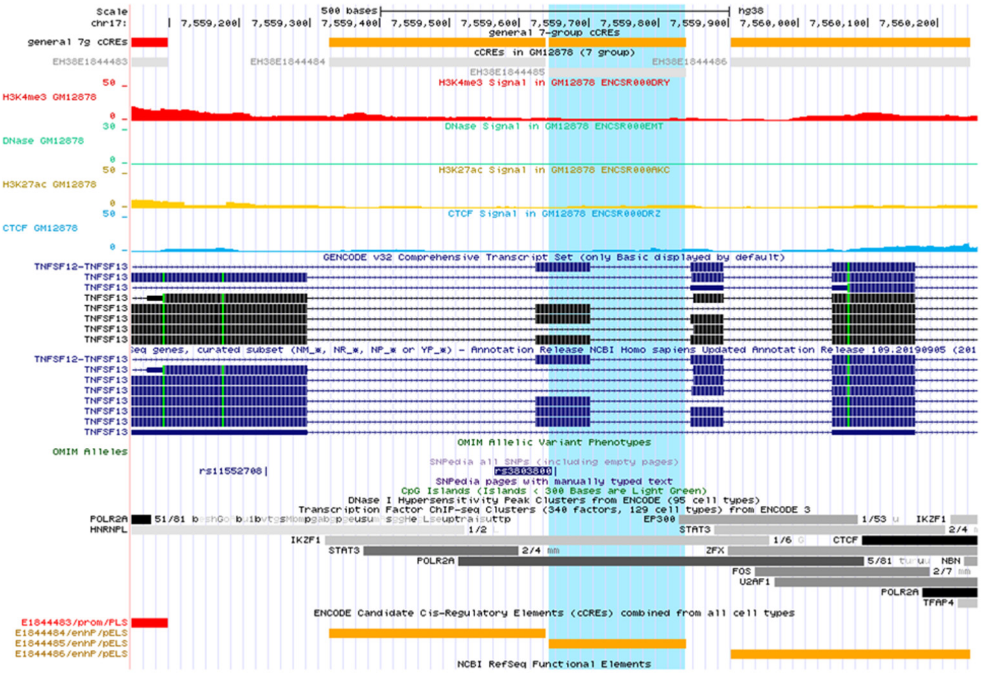

Scale
chr17:
500 bases
hg38
7,559,200
7,559,300
7,559,400
7,559,500
7,559,600
7,559,700
7,559,800
7,559,900
7,560,000
7,560,100
7,560,200
general 7g cCREs
general 7-group cCREs
cCREs in GM12878 (7 group)
EH38E1844483
EH38E1844484
EH38E1844485
EH38E1844486
H3K4me3 GM12878
H3K4me3 Signal in GM12878 ENCSR000DRY
DNase GM12878
DNase Signal in GM12878 ENCSR000EMT
H3K27ac GM12878
H3K27ac Signal in GM12878 ENCSR000AKC
CTCF GM12878
CTCF Signal in GM12878 ENCSR000DRZ
GENCODE v32 Comprehensive Transcript Set (only Basic displayed by default)
TNFSF12-TNFSF13
TNFSF13
Seq genes, curated subset (NM_*, NR_*, NP_* or YP_*) - Annotation Release NCBI Homo sapiens Updated Annotation Release 109.20190905 (201
OMIM Allelic Variant Phenotypes
OMIM Alleles
SNPedia all SNPs (including empty pages)
rs11552708
rs3803800
SNPedia pages with manually typed text
CpG Islands (Islands < 300 Bases are Light Green)
DNase I Hypersensitivity Peak Clusters from ENCODE (95 cell types)
Transcription Factor ChIP-seq Clusters (340 factors, 129 cell types) from ENCODE 3
POLR2A 51/81
HNRNPL 1/2
IKZF1 1/6
STAT3 2/4
POLR2A 5/81
EP300 1/53
IKZF1
STAT3 2/4
CTCF
ZFX
NBN
FOS 2/7
U2AF1
POLR2A
TFAP4
ENCODE Candidate Cis-Regulatory Elements (cCREs) combined from all cell types
E1844483/prom/PLS
E1844484/enhP/pELS
E1844485/enhP/pELS
E1844486/enhP/pELS
NCBI RefSeq Functional Elements

D

Tissue	Samples	NES	p-value	m-value
Esophagus - Mucosa	497	0.0438	0.2	0.00
Liver	208	0.0294	0.7	0.464
Spleen	227	0.0215	0.7	0.311
Small Intestine - Terminal Ileum	174	0.0187	0.8	0.502
Ovary	167	0.0181	0.8	0.619
Minor Salivary Gland	144	0.0125	0.8	0.317
Cells - EBV-transformed lymphocytes	147	-0.00538	1	0.661
Colon - Sigmoid	318	-0.0109	0.8	0.211
Adrenal Gland	233	-0.0152	0.8	0.418
Cells - Cultured fibroblasts	483	-0.0248	0.4	0.0980
Esophagus - Muscularis	465	-0.0258	0.5	0.219
Adipose - Visceral (Omentum)	469	-0.0279	0.5	0.347
Breast - Mammary Tissue	396	-0.0564	0.2	0.857
Colon - Transverse	368	-0.0579	0.08	0.849
Artery - Aorta	387	-0.0585	0.1	0.799
Stomach	324	-0.0670	0.1	0.882
Whole Blood	670	-0.0675	9.5e-4	0.961
Brain - Spinal cord (cervical c-1)	126	-0.0697	0.3	0.771
Artery - Coronary	213	-0.0705	0.2	0.906
Pancreas	305	-0.0854	0.1	0.950
Skin - Not Sun Exposed (Suprapubic)	517	-0.0873	0.009	0.970
Muscle - Skeletal	706	-0.0892	0.02	0.970
Artery - Tibial	584	-0.0956	0.008	0.986
Brain - Substantia nigra	114	-0.0983	0.2	0.854
Thyroid	574	-0.103	0.02	0.978
Pituitary	237	-0.104	0.05	0.979
Heart - Atrial Appendage	372	-0.105	0.01	0.998
Kidney - Cortex	73	-0.106	0.4	0.851
Lung	515	-0.108	1.2e-4	1.00
Brain - Cerebellum	209	-0.109	0.03	0.954
Testis	322	-0.110	0.02	0.979
Skin - Sun Exposed (Lower leg)	605	-0.110	1.2e-3	1.00
Esophagus - Gastroesophageal Junction	330	-0.112	0.01	0.975
Nerve - Tibial	532	-0.115	7.4e-4	1.00
Adipose - Subcutaneous	581	-0.123	3.4e-5	1.00
Heart - Left Ventricle	386	-0.126	0.006	1.00
Brain - Cerebellar Hemisphere	175	-0.130	0.04	0.953
Brain - Hippocampus	165	-0.150	0.007	0.980
Brain - Putamen (basal ganglia)	170	-0.157	8.3e-5	1.00
Brain - Nucleus accumbens (basal ganglia)	202	-0.159	7.6e-5	1.00
Brain - Frontal Cortex (BA9)	175	-0.167	2.6e-5	1.00
Brain - Hypothalamus	170	-0.184	2.1e-3	0.991
Brain - Anterior cingulate cortex (BA24)	147	-0.188	5.7e-5	1.00
Brain - Cortex	205	-0.203	2.5e-8	1.00
Vagina	141	-0.205	0.06	0.917
Prostate	221	-0.217	1.7e-3	0.984
Brain - Caudate (basal ganglia)	194	-0.220	7.8e-7	1.00
Uterus	129	-0.246	0.03	0.946
Brain - Amygdala	129	-0.263	1.2e-4	0.998

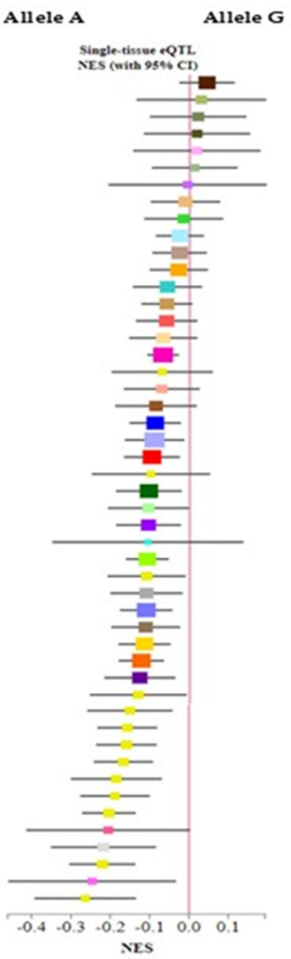

Supplementary Figure S2. *In silico* analysis - rs3803800 of *TNFSF13*.

(A) The result of analysis performed with application of HaploReg v4.1 tool (https://pubs.broadinstitute.org/mammals/haploreg/haploreg.php) (screen shot) [1-2] (B) Location of rs3803800 (highlighted) of TNFSF13 gene in relation to data from ENCODE project showing potential regulatory element-binding sites upstream and downstream of rs3803800 in B cells and GM12878 cell lines (**C**). Figures were downloaded from the UCSC genome browser (http://genome.ucsc.edu) [8-10. A view of the region surrounding the rs3803800 includes also the representation of the ChIP-seq data from the ENCODE project, representing transcription factors (TFs) binding sites (black boxes denote the sites with stronger evidence for TFs binding in that region) (**D**) GTEx multi-tissue eQTLs analysis of association between rs3803800 and *TNFSF13* expression (**Meta-Analysis RE2: p-value=5.2x10^{-55}**).

A

Query SNP: rs4968210 and variants with r^2 >= 0.8

chr	pos (hg38)	LD (r^2)	LD (D')	variant	Ref	Alt	AFR freq	AMR freq	ASN freq	EUR freq	SiPhy cons	Promoter histone marks	Enhancer histone marks	DNAse	Proteins bound	Motifs changed	NHGRI/EBI GWAS hits	GRASP QTL hits	Selected eQTL hits	GENCODE genes	dbSNP func annot
17	7538942	0.8	0.95	rs4968216	G	A	0.02	0.33	0.27	0.40			5 tissues	BRN,LNG	CFOS	LBP-1,LBP-9			21 hits	1.7kb 5' of Y_RNA	
17	7541685	0.82	0.97	rs4968221	G	A	0.12	0.33	0.27	0.40						9 altered motifs			19 hits	4.5kb 5' of Y_RNA	
17	7542075	0.87	0.95	rs148852655	A	20-mer	0.25	0.47	0.47	0.42						YY1			16 hits	4.9kb 5' of Y_RNA	
17	7552484	0.88	1	rs12942590	C	G	0.02	0.32	0.28	0.40			5 tissues			4 altered motifs			22 hits	TNFSF12	intronic
17	7554035	1	1	rs4968210	G	A	0.45	0.51	0.46	0.43		BLD	8 tissues	CRVX	GR	CCNT2,HP1-site-factor		5 hits	20 hits	TNFSF12	intronic
17	7554376	0.85	0.99	rs11871455	C	T	0.14	0.33	0.28	0.39			BLD			E2F,Maf,SREBP			20 hits	TNFSF12	intronic
17	7555265	0.83	-0.99	rs12937543	A	T	0.23	0.45	0.41	0.53			BLD, SKIN					3 hits	14 hits	TNFSF12	intronic
17	7557368	0.87	0.99	rs1128963	G	A	0.02	0.32	0.28	0.40		5 tissues	17 tissues	8 tissues	RAD21	SREBP		1 hit	23 hits	TNFSF12	3'-UTR

B

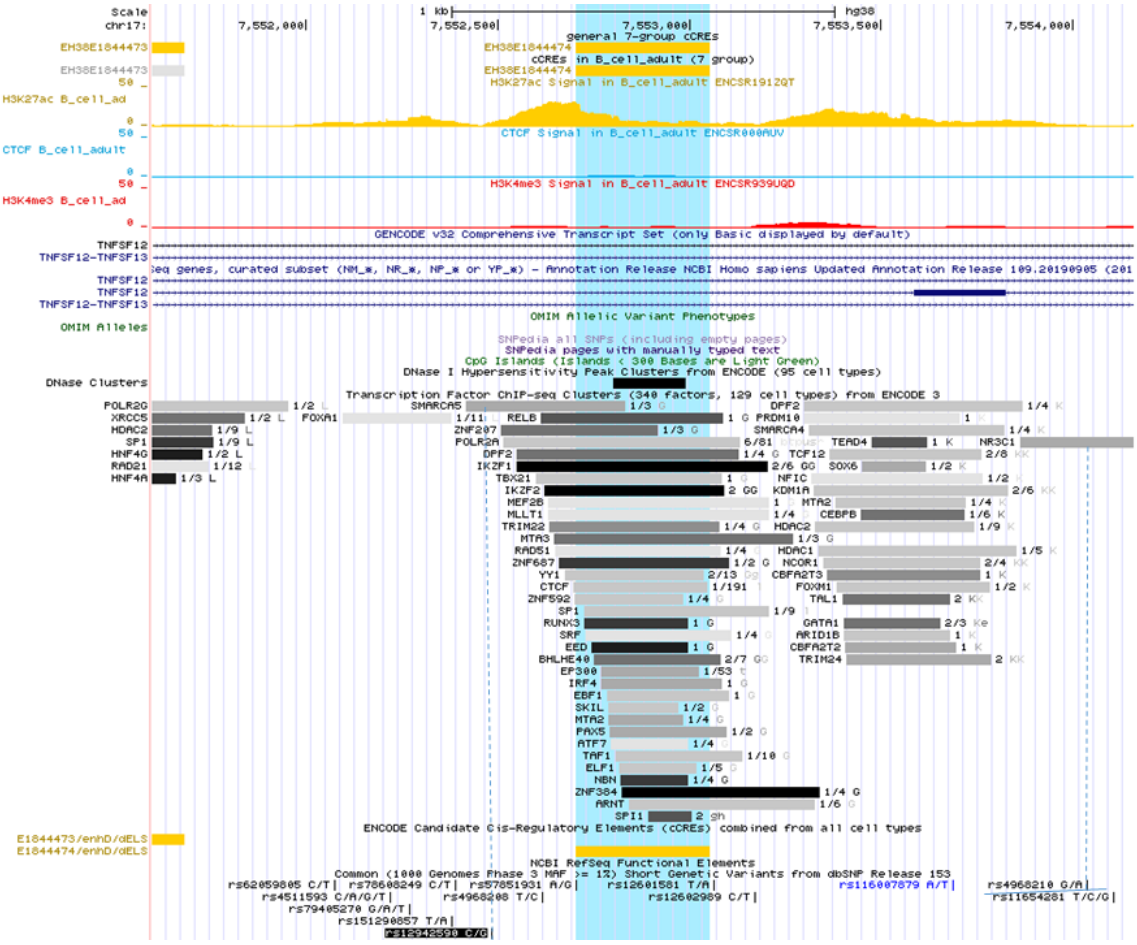

C

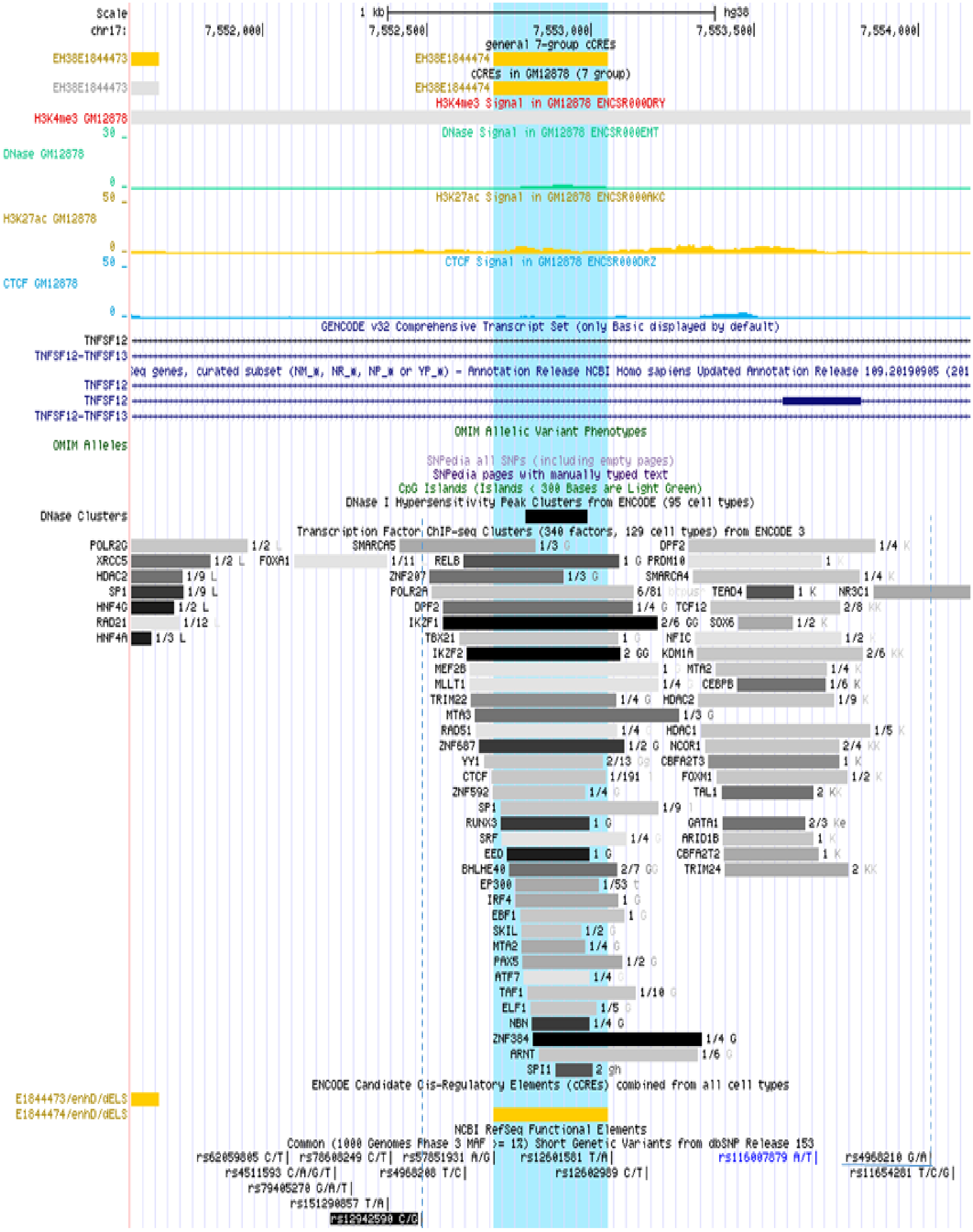

Scale
chr17:
1 kb
hg38
7,552,000
7,552,500
7,553,000
7,553,500
7,554,000
general 7-group cCREs
EH38E1844473
EH38E1844474
cCREs in GM12878 (7 group)
H3K4me3 Signal in GM12878 ENCSR000DRY
H3K4me3 GM12878
DNase Signal in GM12878 ENCSR000EMT
DNase GM12878
H3K27ac Signal in GM12878 ENCSR000AKC
H3K27ac GM12878
CTCF Signal in GM12878 ENCSR000DRZ
CTCF GM12878
GENCODE v32 Comprehensive Transcript Set (only Basic displayed by default)
TNFSF12
TNFSF12-TNFSF13
OMIM Allelic Variant Phenotypes
OMIM Alleles
SNPedia all SNPs (including empty pages)
SNPedia pages with manually typed text
CpG Islands (Islands < 300 Bases are Light Green)
DNase I Hypersensitivity Peak Clusters from ENCODE (95 cell types)
DNase Clusters
Transcription Factor ChIP-seq Clusters (340 factors, 129 cell types) from ENCODE 3
ENCODE Candidate Cis-Regulatory Elements (cCREs) combined from all cell types
E1844473/enhD/dELS
E1844474/enhD/dELS
NCBI RefSeq Functional Elements
Common (1000 Genomes Phase 3 MAF >= 1%) Short Genetic Variants from dbSNP Release 153

D

Tissue	Samples	NES	p-value	m-value
Liver	208	0.127	0.04	0.0130
Kidney - Cortex	73	0.0664	0.5	0.240
Cells - EBV-transformed lymphocytes	147	0.0204	0.8	0.345
Muscle - Skeletal	706	0.0112	0.7	0.00600
Artery - Coronary	213	-0.000239	1	0.0980
Minor Salivary Gland	144	-0.00655	0.9	0.325
Brain - Cerebellar Hemisphere	175	-0.00672	0.9	0.159
Pancreas	305	-0.00788	0.8	0.100
Pituitary	237	-0.0120	0.6	0.00
Testis	322	-0.0186	0.4	0.0330
Thyroid	574	-0.0223	0.3	0.00
Artery - Aorta	387	-0.0269	0.4	0.102
Stomach	324	-0.0272	0.3	0.0590
Ovary	167	-0.0308	0.5	0.399
Small Intestine - Terminal Ileum	174	-0.0311	0.2	0.0740
Colon - Transverse	368	-0.0342	0.04	0.00700
Brain - Cerebellum	209	-0.0402	0.3	0.488
Spleen	227	-0.0436	0.3	0.502
Lung	515	-0.0445	0.04	0.401
Brain - Caudate (basal ganglia)	194	-0.0449	0.1	0.554
Vagina	141	-0.0461	0.2	0.628
Brain - Nucleus accumbens (basal ganglia)	202	-0.0480	0.1	0.551
Brain - Spinal cord (cervical c-1)	126	-0.0500	0.4	0.620
Heart - Left Ventricle	386	-0.0527	0.02	0.732
Heart - Atrial Appendage	372	-0.0547	0.02	0.784
Brain - Cortex	205	-0.0564	0.1	0.763
Brain - Substantia nigra	114	-0.0636	0.2	0.698
Esophagus - Mucosa	497	-0.0692	9.1e-5	1.00
Prostate	221	-0.0697	0.01	0.937
Whole Blood	670	-0.0708	0.007	0.979
Adrenal Gland	233	-0.0711	0.04	0.892
Brain - Putamen (basal ganglia)	170	-0.0724	0.03	0.967
Skin - Sun Exposed (Lower leg)	605	-0.0777	5.6e-6	1.00
Breast - Mammary Tissue	396	-0.0823	2.0e-4	1.00
Skin - Not Sun Exposed (Suprapubic)	517	-0.0896	4.2e-6	1.00
Nerve - Tibial	532	-0.0936	1.8e-7	1.00
Brain - Hypothalamus	170	-0.100	0.03	0.949
Cells - Cultured fibroblasts	483	-0.100	2.6e-5	1.00
Artery - Tibial	584	-0.104	1.5e-7	1.00
Brain - Frontal Cortex (BA9)	175	-0.115	1.8e-3	0.985
Adipose - Visceral (Omentum)	469	-0.122	3.8e-9	1.00
Adipose - Subcutaneous	581	-0.134	7.5e-11	1.00
Brain - Hippocampus	165	-0.147	1.3e-4	1.00
Brain - Anterior cingulate cortex (BA24)	147	-0.151	1.2e-3	1.00
Uterus	129	-0.182	1.7e-3	1.00
Colon - Sigmoid	318	-0.183	8.7e-11	1.00
Esophagus - Gastroesophageal Junction	330	-0.187	4.1e-9	1.00
Brain - Amygdala	129	-0.198	1.2e-3	1.00
Esophagus - Muscularis	465	-0.213	5.9e-22	1.00

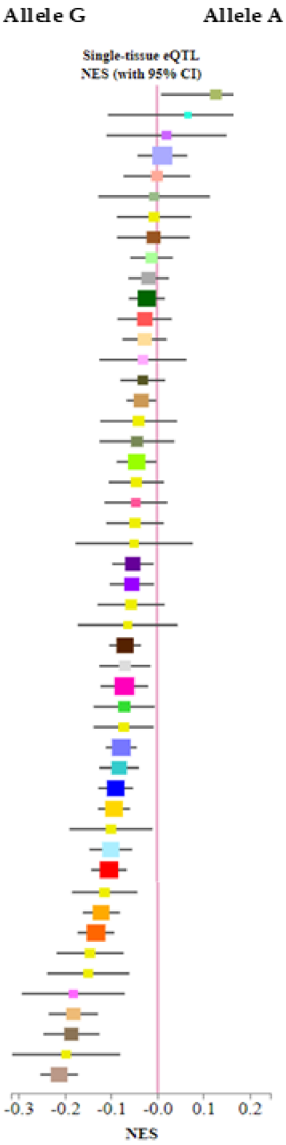

E

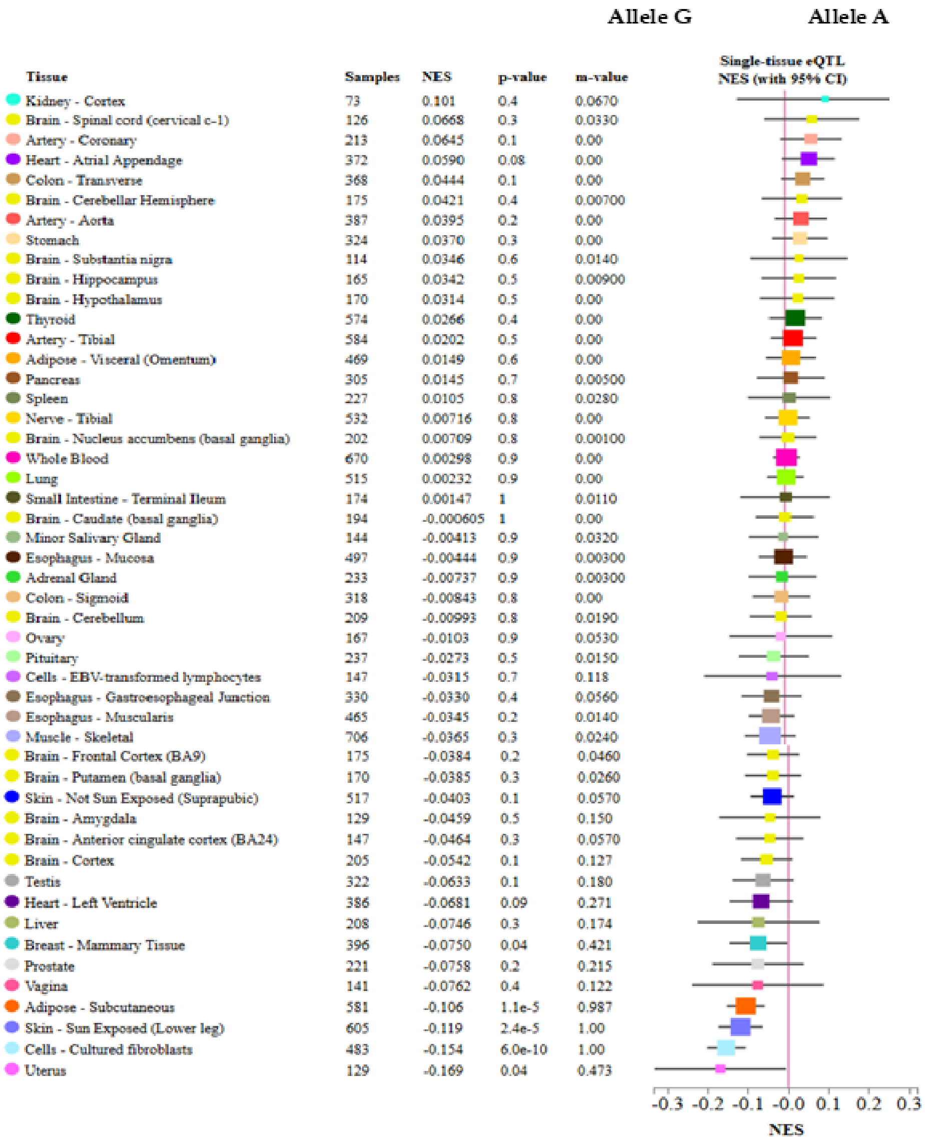

Tissue	Samples	NES	p-value	m-value
Kidney - Cortex	73	0.101	0.4	0.0670
Brain - Spinal cord (cervical c-1)	126	0.0668	0.3	0.0330
Artery - Coronary	213	0.0645	0.1	0.00
Heart - Atrial Appendage	372	0.0590	0.08	0.00
Colon - Transverse	368	0.0444	0.1	0.00
Brain - Cerebellar Hemisphere	175	0.0421	0.4	0.00700
Artery - Aorta	387	0.0395	0.2	0.00
Stomach	324	0.0370	0.3	0.00
Brain - Substantia nigra	114	0.0346	0.6	0.0140
Brain - Hippocampus	165	0.0342	0.5	0.00900
Brain - Hypothalamus	170	0.0314	0.5	0.00
Thyroid	574	0.0266	0.4	0.00
Artery - Tibial	584	0.0202	0.5	0.00
Adipose - Visceral (Omentum)	469	0.0149	0.6	0.00
Pancreas	305	0.0145	0.7	0.00500
Spleen	227	0.0105	0.8	0.0280
Nerve - Tibial	532	0.00716	0.8	0.00
Brain - Nucleus accumbens (basal ganglia)	202	0.00709	0.8	0.00100
Whole Blood	670	0.00298	0.9	0.00
Lung	515	0.00232	0.9	0.00
Small Intestine - Terminal Ileum	174	0.00147	1	0.0110
Brain - Caudate (basal ganglia)	194	-0.000605	1	0.00
Minor Salivary Gland	144	-0.00413	0.9	0.0320
Esophagus - Mucosa	497	-0.00444	0.9	0.00300
Adrenal Gland	233	-0.00737	0.9	0.00300
Colon - Sigmoid	318	-0.00843	0.8	0.00
Brain - Cerebellum	209	-0.00993	0.8	0.0190
Ovary	167	-0.0103	0.9	0.0530
Pituitary	237	-0.0273	0.5	0.0150
Cells - EBV-transformed lymphocytes	147	-0.0315	0.7	0.118
Esophagus - Gastroesophageal Junction	330	-0.0330	0.4	0.0560
Esophagus - Muscularis	465	-0.0345	0.2	0.0140
Muscle - Skeletal	706	-0.0365	0.3	0.0240
Brain - Frontal Cortex (BA9)	175	-0.0384	0.2	0.0460
Brain - Putamen (basal ganglia)	170	-0.0385	0.3	0.0260
Skin - Not Sun Exposed (Suprapubic)	517	-0.0403	0.1	0.0570
Brain - Amygdala	129	-0.0459	0.5	0.150
Brain - Anterior cingulate cortex (BA24)	147	-0.0464	0.3	0.0570
Brain - Cortex	205	-0.0542	0.1	0.127
Testis	322	-0.0633	0.1	0.180
Heart - Left Ventricle	386	-0.0681	0.09	0.271
Liver	208	-0.0746	0.3	0.174
Breast - Mammary Tissue	396	-0.0750	0.04	0.421
Prostate	221	-0.0758	0.2	0.215
Vagina	141	-0.0762	0.4	0.122
Adipose - Subcutaneous	581	-0.106	1.1e-5	0.987
Skin - Sun Exposed (Lower leg)	605	-0.119	2.4e-5	1.00
Cells - Cultured fibroblasts	483	-0.154	6.0e-10	1.00
Uterus	129	-0.169	0.04	0.473

Supplementary Figure S3. *In silico* analysis - rs4968210 of *TNFSF13*.

(A) The result of analysis performed with application of HaploReg v4.1 tool (screen shot) [1-2]. (**B**) Location of rs4968210 (underlined) and rs12942590 (highlighted) (LD with rs4968210) of *TNFSF13* gene in relation to data from ENCODE project showing potential regulatory element-binding in close proximity to rs4968210 in B cells and (**C**) in GM12878 cell line. A view of the region surrounding the rs4968210 and rs12942590 includes also the representation of the ChIP-seq data from the ENCODE project, representing transcription factors (TFs) binding sites (black boxes denote the sites with stronger evidence for TFs binding in that region). Figures were downloaded from the UCSC genome browser (http://genome.ucsc.edu) [8-10] (**D**) GTEx multi-tissue eQTLs analysis of association between rs4968210 and TNFSF12 expression (**Meta-Analysis RE2: p-value=1.2x10^{-94}**) (E)

GTEx multi-tissue eQTLs analysis of association between rs4968210 and TNFSF13 expression (**Meta -Analysis RE2: p-value=1.9x10^{-11}**).

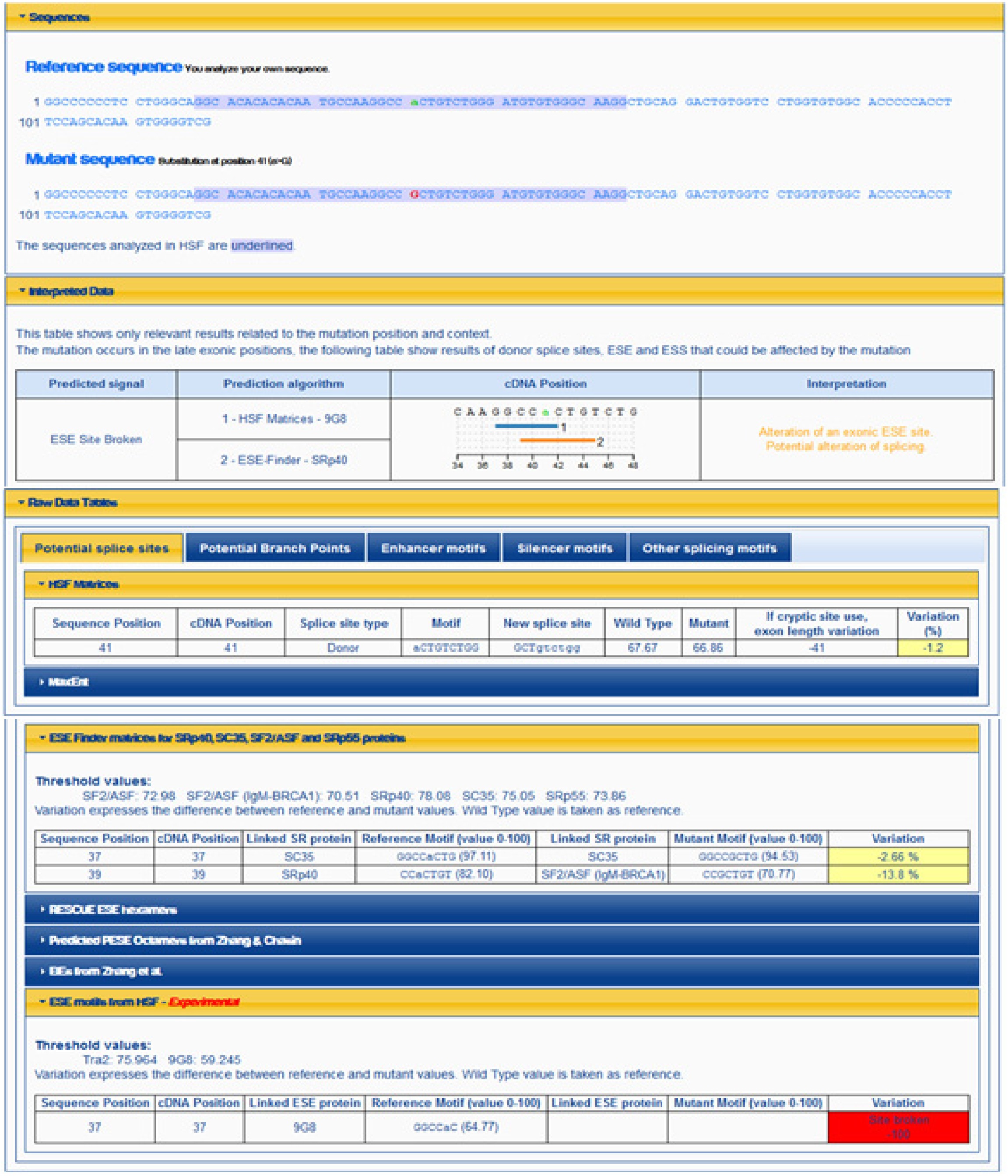

Supplementary Figure S4. Analysis of potential effects of rs11078355 A>G on splicing of *TNFRSF13B* gene. A Human Splicing Finder (HSF version 3.1) [7] prediction showing that allele G of rs11078355 may potentially cause alternation of an exonic ESE site (screen shot).

A

Query SNP: rs11078355 and variants with r^2 >= 0.8

chr	pos (hg38)	LD (r^2)	LD (D')	variant	Ref	Alt	AFR freq	AMR freq	ASN freq	EUR freq	SiPhy cons	Promoter histone marks	Enhancer histone marks	DNAse	Proteins bound	Motifs changed	NHGRI/EBI GWAS hits	GRASP QTL hits	Selected eQTL hits	GENCODE genes	dbSNP func annot
17	16935416	0.97	0.99	rs55898532	A	G	0.63	0.53	0.79	0.36			ADRL, MUS	4 tissues	FOSL2	8 altered motifs			4 hits	TNFRSF13B	
17	16939598	1	1	rs11078355	A	G	0.60	0.55	0.79	0.37			5 tissues	BLD		6 altered motifs			3 hits	TNFRSF13B	synonymous
17	16942841	0.8	0.99	rs12938061	T	C	0.56	0.56	0.79	0.41			4 tissues	10 tissues		9 altered motifs			4 hits	TNFRSF13B	intronic
17	16942862	0.8	0.99	rs12938073	T	A	0.56	0.56	0.79	0.41			4 tissues	9 tissues		4 altered motifs			4 hits	TNFRSF13B	intronic
17	16942984	0.82	0.99	rs11078357	T	C	0.67	0.56	0.79	0.41			6 tissues	BLD,MUS	POL2	Nkx2			3 hits	TNFRSF13B	intronic
17	16943483	0.82	0.99	rs10852841	T	C	0.59	0.55	0.79	0.41						5 altered motifs			3 hits	TNFRSF13B	intronic

B

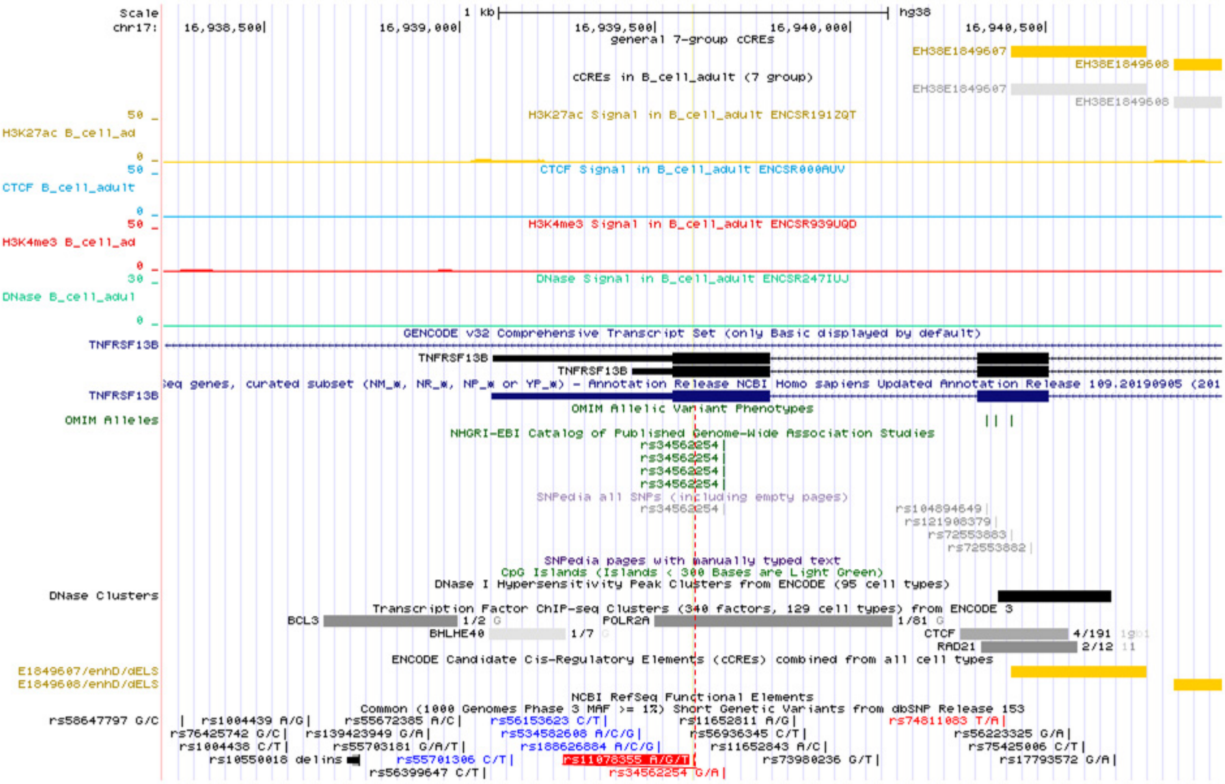

C

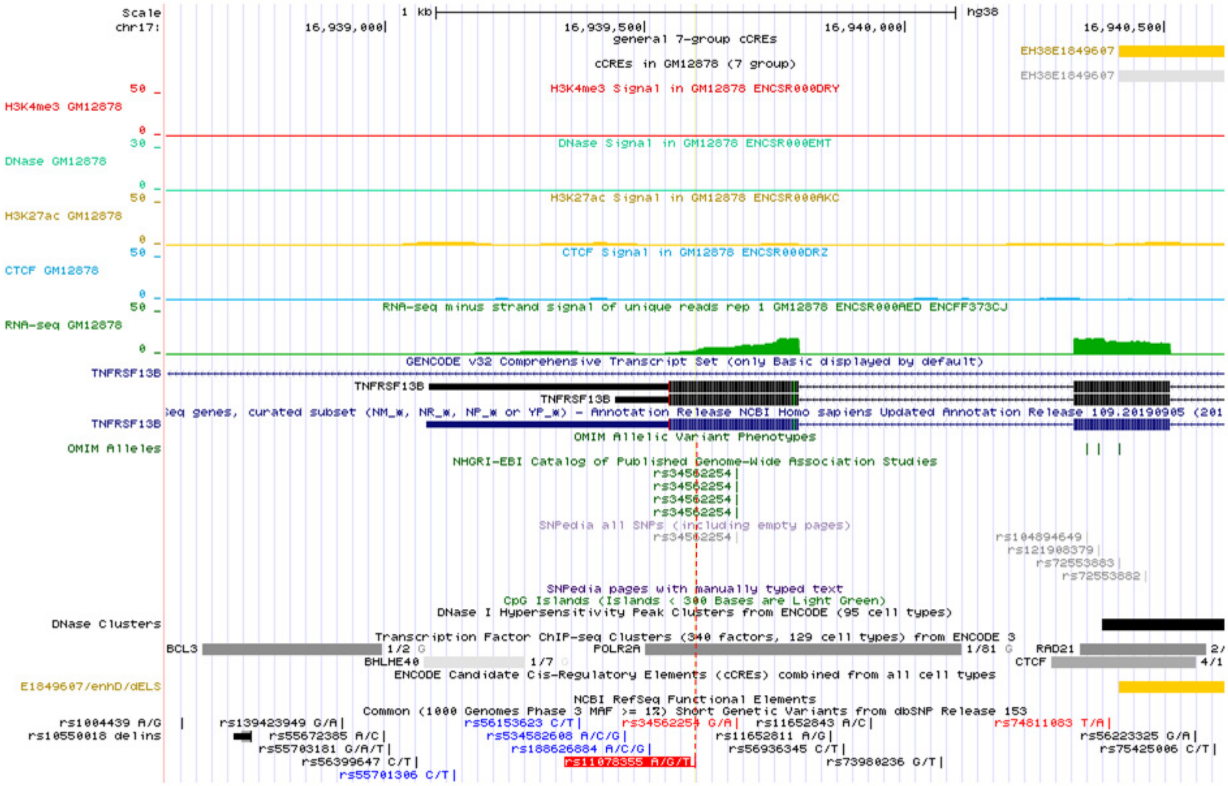

Supplementary Figure S5. *In silico* analysis - rs11078355 of *TNFRSF13B* (**A**) The result of analysis performed with application of HaploReg v4.1 tool (screen shot) [1-2]. Location of rs11078355 of *TNFRSF13B* gene (highlighted) in relation to data from ENCODE project for B cells (**B**) and GM12878 cell line (**D**) showing lack of cCRE in close distance to rs11078355. A view of the region surrounding the rs4968210 and rs12942590 includes also the representation of the ChIP-seq data from the ENCODE project, representing transcription factors (TFs) binding sites (black boxes denote the sites with stronger evidence for TFs binding in that region). Figures were downloaded from the UCSC genome browser (http://genome.ucsc.edu) [8-10].

A

Query SNP: rs4985726 and variants with r^2 >= 0.8

chr	pos (hg38)	LD (r^2)	LD (D')	variant	Ref	Alt	AFR freq	AMR freq	ASN freq	EUR freq	SiPhy cons	Promoter histone marks	Enhancer histone marks	DNAse	Proteins bound	Motifs changed	NHGRI/EBI GWAS hits	GRASP QTL hits	Selected eQTL hits	GENCODE genes	dbSNP func annot
17	16938999	0.8	0.94	rs55672385	A	C	0.11	0.09	0.38	0.11		BLD	6 tissues	THYM,LNG		4 altered motifs				TNFRSF13B	
17	16939677	0.84	0.92	rs34562254	G	A	0.12	0.10	0.39	0.10			5 tissues		POL2	7 altered motifs				TNFRSF13B	missense
17	16941853	0.89	0.97	rs4792800	A	G	0.12	0.10	0.39	0.11			4 tissues	11 tissues		12 altered motifs	1 hit			TNFRSF13B	intronic
17	16945251	0.89	0.97	rs4500785	C	G	0.18	0.11	0.38	0.11			7 tissues	IPSC	POL2	AP-2,NRSF				TNFRSF13B	intronic
17	16945436	0.93	0.97	rs4561508	C	T	0.13	0.10	0.38	0.11			7 tissues			GLI	4 hits			TNFRSF13B	intronic
17	16945825	0.93	0.97	rs4273077	A	G	0.14	0.15	0.47	0.11			8 tissues	20 tissues	CTCF,RAD21	CTCF,Myf,Nkx2	2 hits			TNFRSF13B	intronic
17	16948136	0.93	0.97	rs57382045	G	A	0.13	0.10	0.38	0.11			BLD, BRN	BLD		Brachyury,Msx-1				TNFRSF13B	intronic
17	16951166	0.94	0.97	rs74892229	G	A	0.13	0.10	0.38	0.10				BLD		4 altered motifs				TNFRSF13B	intronic
17	16953548	0.97	0.99	rs57293260	A	T	0.13	0.10	0.38	0.10			BLD, THYM	5 tissues	CTCF	Arid5a				TNFRSF13B	intronic
17	16953863	0.97	0.99	rs57166795	G	A	0.14	0.10	0.38	0.10			BLD, BRN, THYM			7 altered motifs				TNFRSF13B	intronic
17	16960324	1	1	rs4985726	C	G	0.08	0.09	0.38	0.10				BLD			2 hits		1 hit	TNFRSF13B	intronic
17	16963463	0.94	0.97	rs73292853	C	G	0.32	0.17	0.47	0.10			BLD, PANC	IPSC		4 altered motifs				TNFRSF13B	intronic

B

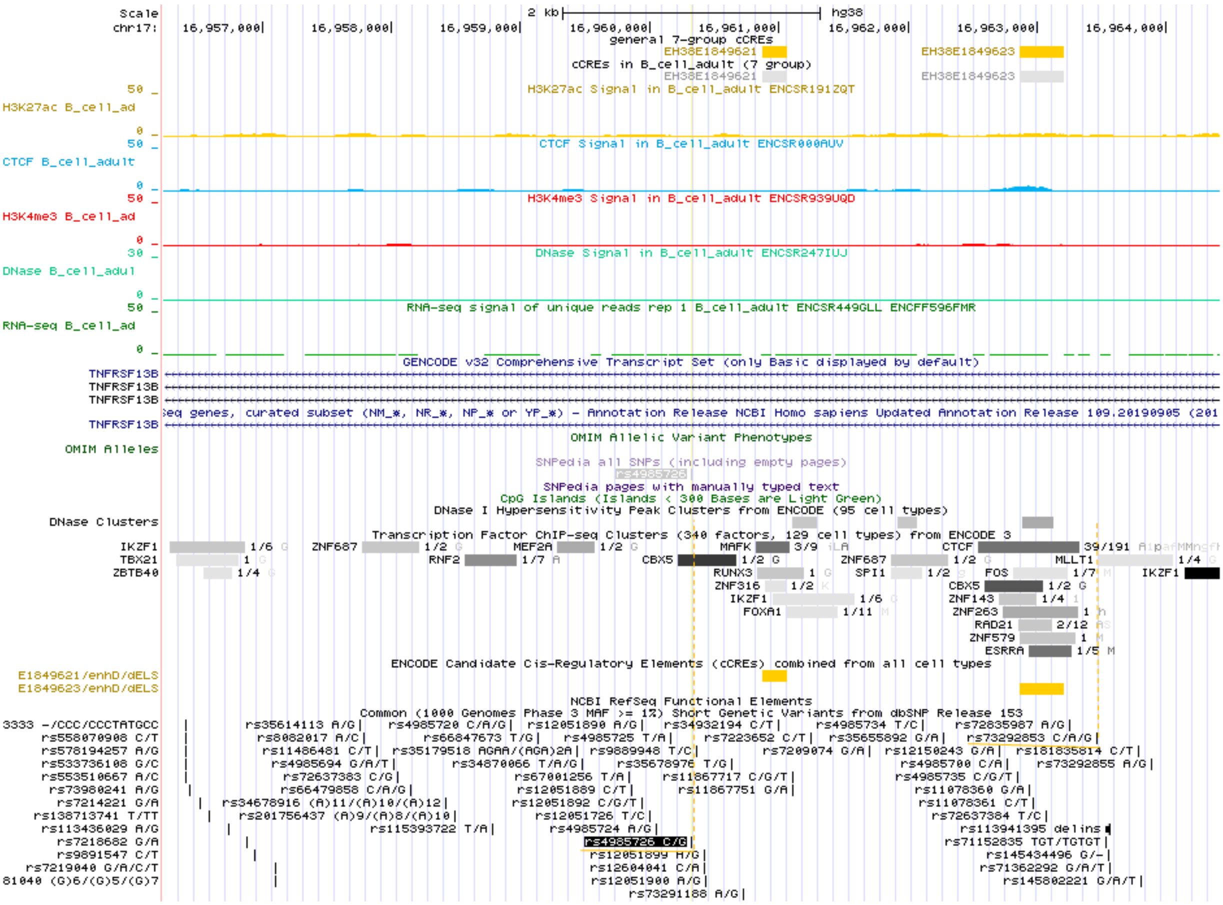

C

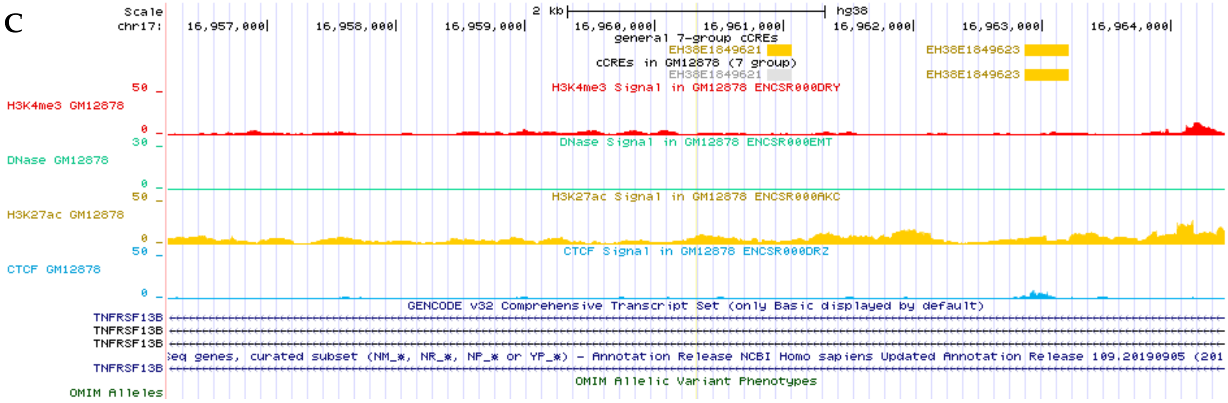
Scale
chr17:
2 kb
hg38
16,957,000| 16,958,000| 16,959,000| 16,960,000| 16,961,000| 16,962,000| 16,963,000| 16,964,000|
general 7-group cCREs
EH38E1849621
EH38E1849623
cCREs in GM12878 (7 group)
EH38E1849621
EH38E1849623
H3K4me3 Signal in GM12878 ENCSR000DRY
H3K4me3 GM12878
DNase Signal in GM12878 ENCSR000EMT
DNase GM12878
H3K27ac Signal in GM12878 ENCSR000AKC
H3K27ac GM12878
CTCF Signal in GM12878 ENCSR000DRZ
CTCF GM12878
GENCODE v32 Comprehensive Transcript Set (only Basic displayed by default)
TNFRSF13B
TNFRSF13B
TNFRSF13B
Seq genes, curated subset (NM_*, NR_*, NP_* or YP_*) - Annotation Release NCBI Homo sapiens Updated Annotation Release 109.20190905 (201
TNFRSF13B
OMIM Allelic Variant Phenotypes
OMIM Alleles

D

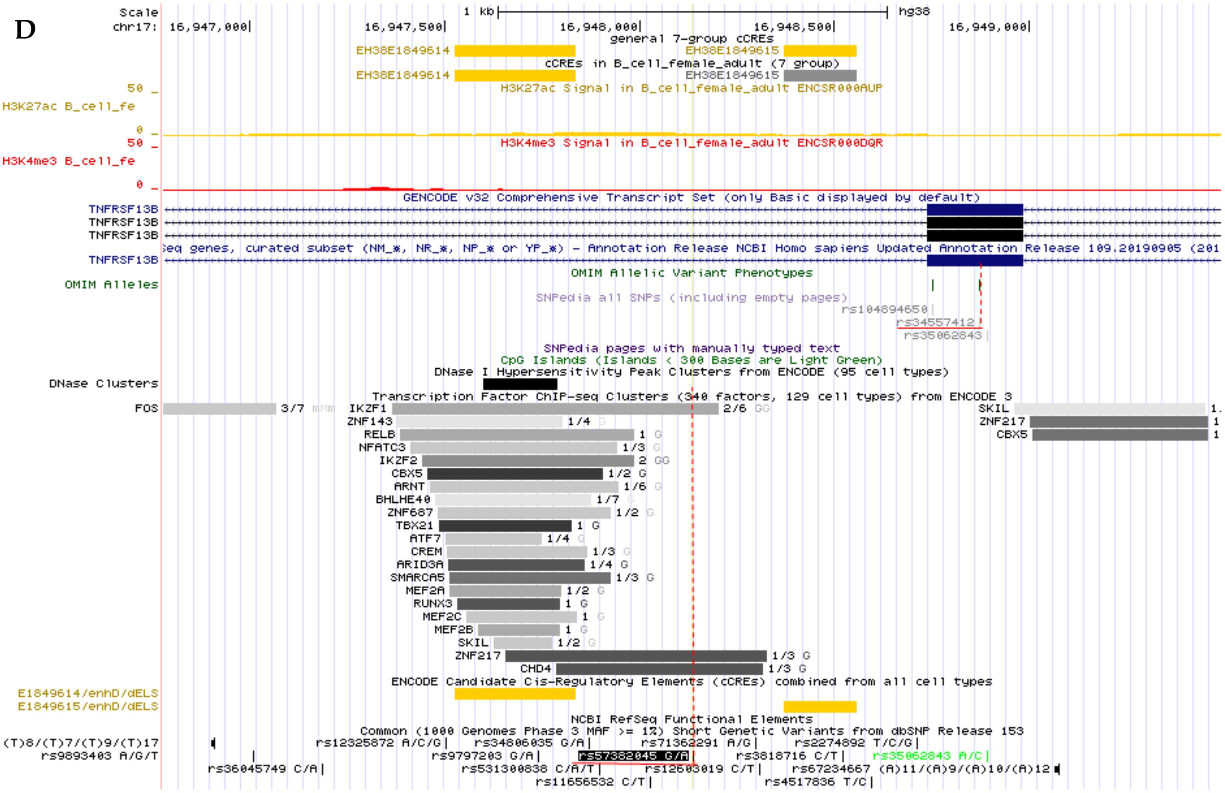
Scale
chr17:
1 kb
hg38
16,947,000| 16,947,500| 16,948,000| 16,948,500| 16,949,000|
general 7-group cCREs
EH38E1849614
EH38E1849615
cCREs in B_cell_female_adult (7 group)
EH38E1849614
EH38E1849615
H3K27ac Signal in B_cell_female_adult ENCSR000AUP
H3K27ac B_cell_fe
H3K4me3 Signal in B_cell_female_adult ENCSR000DQR
H3K4me3 B_cell_fe
GENCODE v32 Comprehensive Transcript Set (only Basic displayed by default)
TNFRSF13B
TNFRSF13B
TNFRSF13B
Seq genes, curated subset (NM_*, NR_*, NP_* or YP_*) - Annotation Release NCBI Homo sapiens Updated Annotation Release 109.20190905 (201
TNFRSF13B
OMIM Allelic Variant Phenotypes
OMIM Alleles
SNPedia all SNPs (including empty pages)
rs104894650
rs34557412
rs35062843
SNPedia pages with manually typed text
CpG Islands (Islands < 300 Bases are Light Green)
DNase I Hypersensitivity Peak Clusters from ENCODE (95 cell types)
DNase Clusters
Transcription Factor ChIP-seq Clusters (340 factors, 129 cell types) from ENCODE 3
FOS 3/7
IKZF1 2/6
SKIL
ZNF217
CBX5
ZNF143 1/4
RELB 1
NFATC3 1/3
IKZF2 2
CBX5 1/2
ARNT 1/6
BHLHE40 1/7
ZNF687 1/2
TBX21 1
ATF7 1/4
CREM 1/3
ARID3A 1/4
SMARCA5 1/3
MEF2A 1/2
RUNX3 1
MEF2C 1
MEF2B 1
SKIL 1/2
ZNF217 1/3
CHD4 1/3
ENCODE Candidate Cis-Regulatory Elements (cCREs) combined from all cell types
E1849614/enhD/dELS
E1849615/enhD/dELS
NCBI RefSeq Functional Elements
Common (1000 Genomes Phase 3 MAF >= 1%) Short Genetic Variants from dbSNP Release 153
(T)8/(T)7/(T)9/(T)17
rs9893403 A/G/T
rs12325872 A/C/G
rs34806035 G/A
rs71362291 A/G
rs2274892 T/C/G
rs9797203 G/A
rs57382045 G/A
rs3818716 C/T
rs35062843 A/C
rs36045749 C/A
rs531300838 C/A/T
rs12603019 C/T
rs67234667 (A)11/(A)9/(A)10/(A)12
rs11656532 C/T
rs4517836 T/C

E

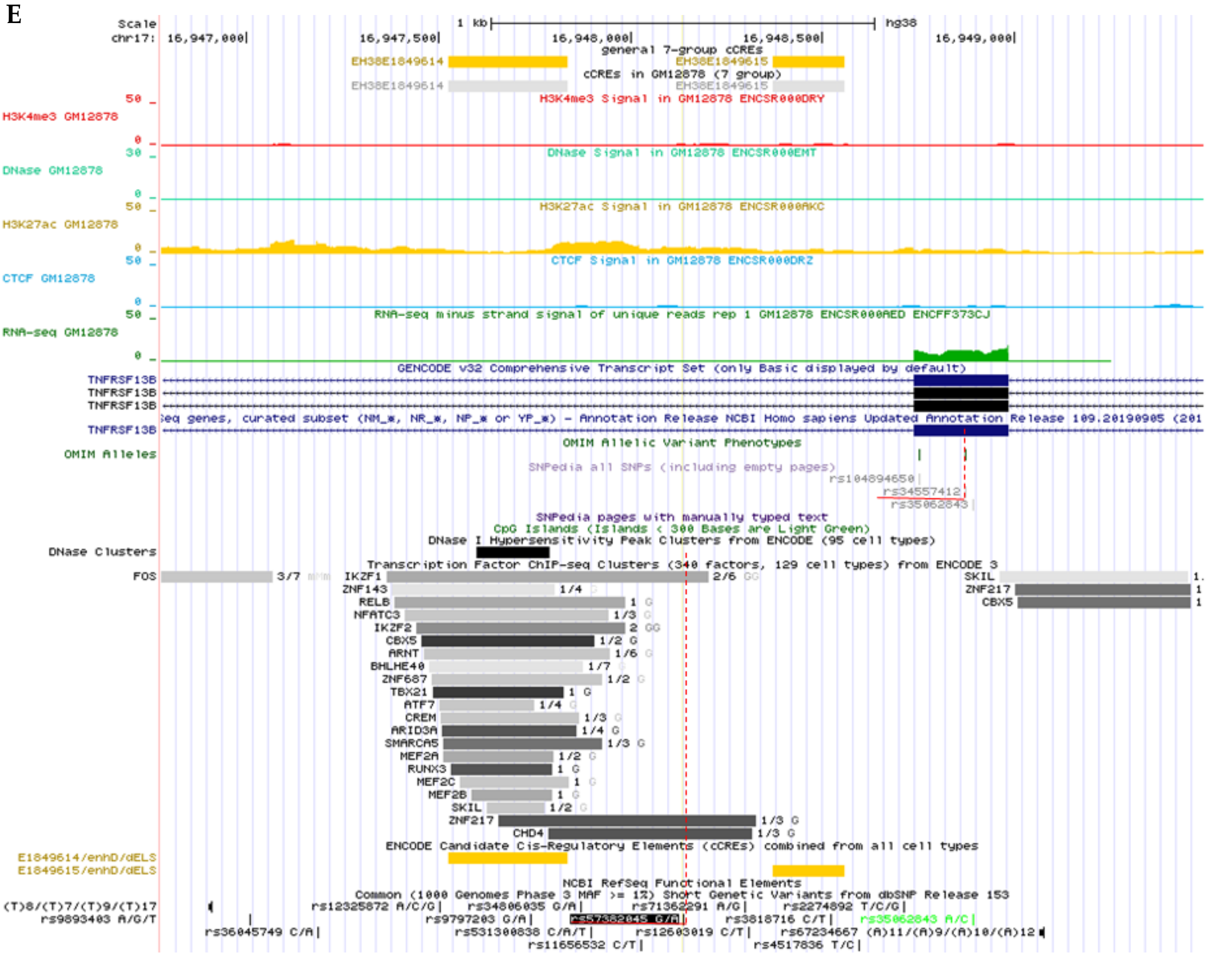

Supplementary Figure S6. *In silico* analysis - rs4985726 of *TNFRSF13B* (**A**) The result of analysis performed with application of HaploReg v4.1 tool (screen shot) [1-2] (**B**, **D**) Location of rs4985726 (highlighted) and rs57382045 (highlighted) (LD with rs4985726) and of *TNFRSF13B* gene in relation to data from ENCODE project for B cells and (**C**, **E**) GM12878 cell line. The dotted lines indicated SNPs in LD with rs4985726. A view of the region surrounding the rs4968210 and rs12942590 includes also the representation of the ChIP-seq data from the ENCODE project, representing transcription factors (TFs) binding sites (black boxes denote the sites with stronger evidence for TFs binding in that region). Figures were downloaded from the UCSC genome browser (http://genome.ucsc.edu) [8-10].

Supplementary Table S8. Genotype distribution of the *BCMA* (*TNFRSF17;* 16p13.13) polymorphisms in patients and controls.

BCMA ***TNFRSF17*** **polymorphisms**		**Patients (N=439)**		**Controls (N=477)**		**OR**	**CI95%**	**Patients vs. Controls**
		**N**	**%**	**N**	**%**			
**rs11570136** 2KB Upstream Variant	TT	205	46.70	213	44.70	1*		
	TA	185	42.10	204	42.80	0.72	0.68 ;1.24	$\chi^2_{df=2}$ =0.61
	AA	49	11.20	60	12.60	0.85	0.56 ;1.30	p=0.735
**HWE**		p=0.587 *f*=0.035 CI95%=-0.06;0.13		p=0.3032 *f*=0.046 CI95%=-0.04;0.14				
**rs2017662**	GG	386	87.90	421	88.30	1*		$\chi^2_{df=2}$ =2.18
Thr159Thr	GA	51	11.60	56	11.70	0.99	0.66; 1.49	p=0.336
	AA	2	0.50	0	0.00	5.45	0.26; 113.94	
**HWE**		p=0.8217 *f*=0.011 CI95%=0.07;0.12		p=0.1732 *f*=-0.062 CI95%=-0.08;-0.05				
**rs373496**	CC	424	96.60	453	95.00	1*		
Ser81Asn	CT	14	3.20	24	5.00	0.63	0.33 ;1.23	$\chi^2_{df=2}$ =3.14
	TT	1	0.20	0	0.00	3.20	0.13 ;78.89	p=0.2210
**HWE**		p=0.027 *f*=-0.11 CI95%=-0.02;0.35		p=0.5730 *f*=-0.02 CI95%=-0.04;0.02				

rs2017662 GA+AA *vs.* GG (OR=1.03; CI95%=0.69-1.54; p-value=0.8765)

rs373496 CT+TT *vs.* CC (OR=0.68; CI95%=0.35-1.29; p-value=0.2269)

Supplementary Table S9. Basic statistics of the main variables considered in the paper.

**Rai**	**0**	**1**	**2**	**3**	**4**	**Sum**
**n**	73	67	74	12	20	246
%	29.7	27.2	30.1	4.9	8.1	100%
	**Min**	**Q1**	**Median**	**Sn**	**Q3**	**Max**
**IgA [g/l]**	0.13	0.8	1.375	0.82	2.192	127
**sAPRIL [ng/ml]**	0.02	16.8	28.1	15.8	42.75	162.4
**APRIL$^+$ CD19$^+$[%]**	0.79	4.298	8.89	8	19.08	73.74
**APRIL$^+$ CD19$^+$ [MFI]**	30.12	53.47	82.82	38	121.2	226.6
**TACI$^+$ CD19$^+$[%]**	0.18	3.043	10.32	10.1	33.09	92.31
**TACI $^+$CD19$^+$[MFI]**	18.88	28.45	38.35	16.1	58.05	152.8

Q1, Q3 – 1st and 3rd quartile

Sn – variability measure

MFI – Mean Fluorescence Intensit

Supplementary Table S10. Primer sequences, annealing temperatures and restriction enzymes used for PCR-RFLP genotyping.

SNP	Forward primer (5′-3′)	Reverse primer (5′-3′)	T_a	Restriction enzyme
**rs4985726**	TTCTATTTCTTTTTCTTGTCTAAGTGC	TGGTAGTTCCACACTAAAGGAATG	52°C	*DpnII*
**rs8072293**[a]	CATCAGGGACAAGAGGCC	CCTGACTGTGGGGCCAGA	55°C	*BslI*
**rs11078355**	AGCCCTGTGAGCACATCC	TCTTTCTCTCTCCCCTCCTCT	52°C	*AciI*

Ta – annealing temperatures

[a] OneTaq Hot Start 2X Master Mix with GC Buffer (New England BioLabs) was used for PCR

Supplementary Table S11. TaqMan SNP Genotyping Assays used for genotyping with applying the allelic discrimination method.

SNP	ID of TaqMan SNP Genotyping Assays
**rs11552708**	C_25630192_20
**rs3803800**	C_12115389_10
**rs4968210**	C_26603822_10
**rs6608**	C_247220_20
**rs12051889**	C_31002822_20
**rs11656106**	C_12121510_10

References

1. Ward, L. D.; Kellis, M., HaploReg: a resource for exploring chromatin states, conservation, and regulatory motif alterations within sets of genetically linked variants. *Nucleic Acids Res* **2012,** *40* (Database issue), D930-4.
2. Ward, L. D.; Kellis, M., HaploReg v4: systematic mining of putative causal variants, cell types, regulators and target genes for human complex traits and disease. *Nucleic Acids Res* **2016,** *44* (D1), D877-81.
3. Bojarska-Junak, A.; Hus, I.; Chocholska, S.; Wasik-Szczepanek, E.; Sieklucka, M.; Dmoszynska, A.; Rolinski, J., BAFF and APRIL expression in B-cell chronic lymphocytic leukemia: correlation with biological and clinical features. *Leuk Res* **2009,** *33* (10), 1319-27.
4. Davis, C. A.; Hitz, B. C.; Sloan, C. A.; Chan, E. T.; Davidson, J. M.; Gabdank, I.; Hilton, J. A.; Jain, K.; Baymuradov, U. K.; Narayanan, A. K.; Onate, K. C.; Graham, K.; Miyasato, S. R.; Dreszer, T. R.; Strattan, J. S.; Jolanki, O.; Tanaka, F. Y.; Cherry, J. M., The Encyclopedia of DNA elements (ENCODE): data portal update. *Nucleic Acids Res* **2018,** *46* (D1), D794-D801.
5. Consortium, E. P., An integrated encyclopedia of DNA elements in the human genome. *Nature* **2012,** *489* (7414), 57-74.
6. Consortium, G. T., The Genotype-Tissue Expression (GTEx) project. *Nat Genet* **2013,** *45* (6), 580-5.
7. Desmet, F. O.; Hamroun, D.; Lalande, M.; Collod-Beroud, G.; Claustres, M.; Beroud, C., Human Splicing Finder: an online bioinformatics tool to predict splicing signals. *Nucleic Acids Res* **2009,** *37* (9), e67.
8. Lee, C. M.; Barber, G. P.; Casper, J.; Clawson, H.; Diekhans, M.; Gonzalez, J. N.; Hinrichs, A. S.; Lee, B. T.; Nassar, L. R.; Powell, C. C.; Raney, B. J.; Rosenbloom, K. R.; Schmelter, D.; Speir, M. L.; Zweig, A. S.; Haussler, D.; Haeussler, M.; Kuhn, R. M.; Kent, W. J., UCSC Genome Browser enters 20th year. *Nucleic Acids Res* **2020,** *48* (D1), D756-D761.
9. Kent, W. J.; Sugnet, C. W.; Furey, T. S.; Roskin, K. M.; Pringle, T. H.; Zahler, A. M.; Haussler, D., The human genome browser at UCSC. *Genome Res* **2002,** *12* (6), 996-1006.
10. Rosenbloom, K. R.; Sloan, C. A.; Malladi, V. S.; Dreszer, T. R.; Learned, K.; Kirkup, V. M.; Wong, M. C.; Maddren, M.; Fang, R.; Heitner, S. G.; Lee, B. T.; Barber, G. P.; Harte, R. A.; Diekhans, M.; Long, J. C.; Wilder, S. P.; Zweig, A. S.; Karolchik, D.; Kuhn, R. M.; Haussler, D.; Kent, W. J., ENCODE data in the UCSC Genome Browser: year 5 update. *Nucleic Acids Res* **2013,** *41* (Database issue), D56-63.

© 2020 by the authors. Submitted for possible open access publication under the terms and conditions of the Creative Commons Attribution (CC BY) license (http://creativecommons.org/licenses/by/4.0/).
